# Mouse models to study von Willebrand factor in thrombosis: a scoping review

**DOI:** 10.1016/j.rpth.2026.106863

**Published:** 2026-07-15

**Authors:** Veronica DeYoung, Hassan Masood, Peter Andrisani, Arthane Kodeeswaran, Taylor Sparring, Jaskirat Arora, Natasha Savic, Jonathan L. Babulic, Patricia C. Liaw, Davide Matino, Alison Fox-Robichaud, Colin A. Kretz

**Affiliations:** 1 Thrombosis and Atherosclerosis Research Institute (TaARI), Hamilton, Canada; 2 Department of Medicine, Queen’s University, Kingston, Canada; 3 Department of Medicine, Faculty of Health Sciences, McMaster University, Hamilton, Canada; 4 Hamilton Health Sciences, Hamilton, Canada; 5 Division of Critical Care, Department of Medicine, Faculty of Health Sciences, McMaster University, Hamilton, Canada

**Keywords:** ferric chloride, mice, reference standards, thrombosis, von Willebrand factor

## Abstract

**Background:**

von Willebrand factor (VWF) plays a significant role in blood coagulation and inflammation and is a key driver of many thrombotic conditions. Identifying appropriate mouse models is critical to developing effective and safe VWF-targeted therapies.

**Objectives:**

This scoping review aims to identify and evaluate all mouse models of thrombosis that have demonstrated functional involvement of VWF and/or its primary regulator, ADAMTS-13, followed by a critical review of common models.

**Methods:**

A comprehensive literature search was conducted to identify studies published from database inception to October 2024 that used mouse models of thrombosis. Two reviewers independently screened and extracted data on model methodology and outcomes, as well as secondary measures of thrombosis if they pertain directly to VWF or indirectly through ADAMTS-13.

**Results:**

A total of 143 studies published between 2000 and 2024 were included; 32.6% models used C57BL/6 mice, and 32.7% used male mice only. Mice age and weight were reported in 79.7% and 30.8% of studies, respectively. Ferric chloride models were used in 45 (31.5%) studies, and laser injury models in 14 (9.8%). The most common systemic thrombosis model was thrombotic thrombocytopenic purpura (21 studies, 14.7%), and there were 16 other diseases studied with secondary thrombosis outcomes pertaining to VWF.

**Conclusion:**

There is significant methodological heterogeneity across mouse models of thrombosis that have demonstrated involvement of VWF. Lack of standardization negatively impacts cross-study comparability and hinders the strength of preclinical studies of novel therapeutics. Standardization of model methodology is urgently needed, and in the future, researchers should consider the impact of methodology on VWF-related outcomes when designing their studies.

## Essentials

• von Willebrand factor (VWF) helps regulate blood clotting in stroke, heart attack, and other diseases.
• We reviewed mouse studies of blood clotting linked to VWF or its regulator, ADAMTS-13.
• 143 mouse studies were included, with varying methodology and reporting of important variables.
• Poor study standardization may limit reliability of research and slow new treatment development.

## 1 Introduction

von Willebrand factor (VWF) is a large multimeric glycoprotein that plays a central role in hemostasis by serving as the carrier for coagulation factor VIII and mediating platelet adhesion at sites of vascular injury [1,2]. Beyond its canonical hemostatic functions, VWF contributes to vascular inflammation by facilitating the recruitment of inflammatory cells to injured endothelium, positioning it as a key mediator at the intersection of thrombosis and inflammation [3]. The protease ADAMTS-13 (a disintegrin and metalloproteinase with a thrombospondin type 1 motif, member 13) regulates VWF activity through shear-dependent cleavage of ultra-large, highly prothrombotic VWF multimers, preventing the formation of occlusive thrombi [4]. Disruption of the ADAMTS-13–VWF axis has been implicated in a wide range of thromboinflammatory conditions, including stroke [5], myocardial infarction [6], kidney disease [7], sepsis [8], colitis [9], and thrombotic thrombocytopenic purpura (TTP) [10], among others [[11], [12], [13], [14]]. A recent Mendelian randomization analysis further supports a causal role for VWF in venous thromboembolism, peripheral artery disease, coronary artery disease, myocardial infarction, and ischemic stroke [15].

Current antithrombotic therapies primarily target coagulation pathways, platelet activation, or fibrinolysis [16]. In contrast, therapeutic strategies targeting VWF represent a relatively recent but rapidly expanding area of investigation. Recombinant ADAMTS-13 is now approved for treatment of congenital TTP [17] and under evaluation for acquired TTP [18], while caplacizumab—an anti-VWF nanobody that prevents platelet binding—is approved for use in acute TTP [19]. Interest is growing in extending VWF-targeted approaches to other thrombotic diseases. Much of the rationale for these efforts stems from preclinical studies using mouse models of thrombosis to assess the contribution of VWF and ADAMTS-13 to thrombus formation and resolution.

Numerous mouse models of thrombosis have been developed, each activating distinct pathogenic mechanisms and producing thrombi with unique cellular and molecular compositions. Choice of model often depends on biological relevance, technical feasibility, and the specific pathway of interest [20]. For example, ferric chloride-induced arterial injury causes endothelial denudation and promotes aggregation of intravascular proteins [21], producing VWF- and platelet-rich thrombi [22,23]. These features are relevant to diseases such as atherosclerotic plaque rupture [24]. In contrast, venous stenosis or ligation models produce stasis-driven thrombi enriched in fibrin and red blood cells (RBCs) [25], better reflecting the pathogenesis of deep vein thrombosis [26]. These pathophysiologic differences have translational implications: for example, tissue plasminogen activator (tPA) is less effective against platelet- and VWF-rich thrombi than against RBC- and fibrin-rich thrombi in patients [27], emphasizing the importance of selecting models that accurately reproduce the thrombus phenotype relevant to a given therapeutic target.

Currently, models of thrombosis are not standardized across institutions, although efforts have been made to address this [20,[28], [29], [30]]. Fortunately, a subcommittee on models of thrombosis and hemostasis of the International Society on Thrombosis and Hemostasis Scientific and Standardization Committee states that a project focused on developing standardized protocols is currently underway, with projected completion in 2027 [31]. Despite the breadth of available thrombosis models, no comprehensive resource currently exists that systematically identifies mouse models in which VWF and/or ADAMTS-13 play a demonstrated mechanistic role. To address this gap, we conducted a scoping review designed to transparently and comprehensively identify all mouse thrombosis models with evidence of VWF and/or ADAMTS-13 involvement. We critically evaluated these models to determine their suitability for studying VWF-mediated thrombotic mechanisms and identified unifying concepts for future studies. This work aims to provide investigators with a consolidated resource for model selection, highlight gaps in current knowledge, and inform the development of VWF-targeted therapeutics for thrombotic disease.

## 2 Methods

### 2.1 Protocol and registration

This scoping review adheres to the Preferred Reporting Items for Systematic reviews and Meta-Analyses extension for Scoping Reviews (PRISMA-ScR) [32]. The study was a retrospective analysis of the literature, and therefore, approval from an ethics board was not required. The protocol for this study was published in Open Science Framework and can be accessed with the identifier: https://doi.org/10.17605/OSF.IO/N8VC7.

### 2.2 Research question

What are the mouse models that have demonstrated mechanistic involvement of VWF in the context of thrombosis?

### 2.3 Search strategy

A literature search was conducted using MEDLINE, Web of Science, and EMBASE from inception to October 2024 to identify mouse models of inflammation, thrombosis, and hemostasis that were used to study VWF. The primary search terms were von Willebrand Factor, VWF, ADAMTS13, ADAMTS-13, mice, and mouse. Associated subheadings were selected and terms were expanded where available in each database (see example in Supplementary Material).

### 2.4 Eligibility criteria and selection process

Using Covidence (Veritas Health Innovation), all full citations from the search were uploaded, and a trial with 25 randomly selected studies was performed to pilot the screening process for both title-abstract and full-text stages. During this process, conflicts were discussed and the inclusion and exclusion criteria were refined accordingly. Two independent reviewers then performed title-abstract screening for relevance, followed by full-text review of the remaining studies. Reviewers were blinded to the responses of other reviewers when choosing to include or exclude citations. Conflicts were resolved through discussion or input from a third reviewer. Reasons for exclusion at the full-text stage were recorded, and deduplication was performed automatically through Covidence, or manually for any that were missed.

Inclusion criteria: uses mouse model(s) of thrombosis or of a disease state with secondary thrombosis measures; direct *in vivo* outcome measure showing VWF or ADAMTS-13 involvement in the model; primary study.

Exclusion criteria: unavailable in English; full-text unavailable; species other than mouse; VWF used only as a marker for a process unrelated to the aims of this review (eg, identifying endothelial cells in immunohistological stains); no model of thrombosis or a disease state with relevant secondary measures; model of angiogenesis; outcome measures only obtained *in vitro* or *ex vivo*; no direct *in vivo* outcome measure showing involvement of VWF or ADAMTS-13; outcome only quantifies gene expression; editorials, abstracts, commentaries, letters, systematic reviews, and meta-analyses.

### 2.5 Data extraction

Key information was extracted from the included studies, including:

1. Author(s)
2. Title
3. Year of publication
4. Country of publication
5. Study objective (eg, therapeutic, mechanistic, diagnostic, etc.)
6. Model or outcome type(s) reported (ie, thrombosis, hemostasis, inflammation)
7. Animal characteristics (eg, sex, weight, age, source)
8. Model methodology, including humane endpoints and relevant agent dosing and administration
9. Interventions and comparators, including dosing, if relevant for understanding associated outcome measures
10. Outcomes involving VWF or ADAMTS-13 (eg, clinical signs, plasma levels, protein activity, immunostaining, etc.) and associated relevant methodology

Data extraction was conducted asynchronously in Excel (Microsoft) using a template developed by the group a priori. The extraction form was first pilot tested using 20 studies extracted by 2 independent reviewers, and modifications were made within the template accordingly. Each study had data extracted by 2 independent reviewers, with discrepancies resolved through discussion or third-party arbitration afterward. Interrater reliability was ensured by having a minimum of 20% overlap of included studies independently reviewed and compared.

For this study, extracted data was filtered in Excel using the ‘thrombosis’ tag to identify all studies that used a model of thrombosis or used a model with secondary measures of thrombosis. The remaining studies identified by these methods (ie, inflammation and hemostasis models) will be published in separate reviews.

## 3 Results

### 3.1 Study selection

The study selection process is depicted in Figure 1. The search strategy returned 5279 unique citations after deduplication. Following title-abstract and full-text screening, 258 articles were selected for data extraction pertaining to inflammation, thrombosis, and/or hemostasis. For the purposes of this scoping review, all studies utilizing thrombosis models or measuring thrombosis-related outcomes were included (*n* = 143).

**Figure 1:**
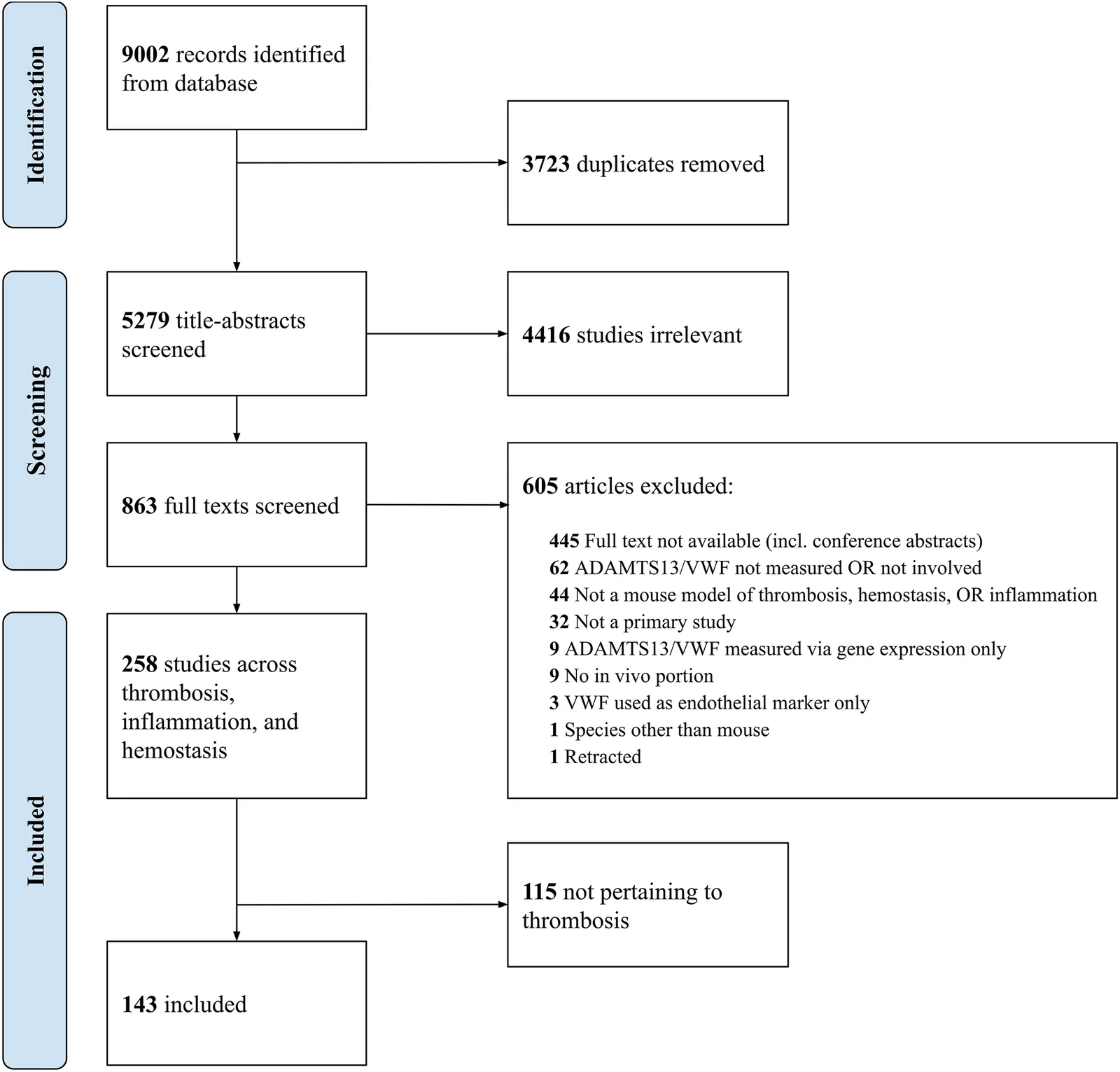
Preferred Reporting Items for Systematic Reviews and Meta-Analyses (PRISMA) flow chart. ADAMTS-13, a disintegrin and metalloproteinase with a thrombospondin type 1 motif, member 13; VWF, von Willebrand factor.

### 3.2 Study characteristics

The characteristics of the 143 included studies are summarized in Figure 2. Studies were published between 2000 and 2024 and were most often conducted in the United States (44.1%). The Jackson Laboratory was the most common source of experimental mice (28.0% of 151 total sources), and 32.9% of studies used male mice only. Of note, 20.3% of studies did not report the age of the mice, and 69.2% of studies did not report the weight of the mice at the beginning of experiments. The most common strain of mice reported was C57BL/6J and C57BL/6. The ‘other’ strains group included C57BL/6NCrl, BALB/c, Kunming, ddY, Berkeley, CD1, OF1, FVB, and Swiss. All included studies and their characteristics are outlined in Supplementary Table 1.

**Figure 2:**
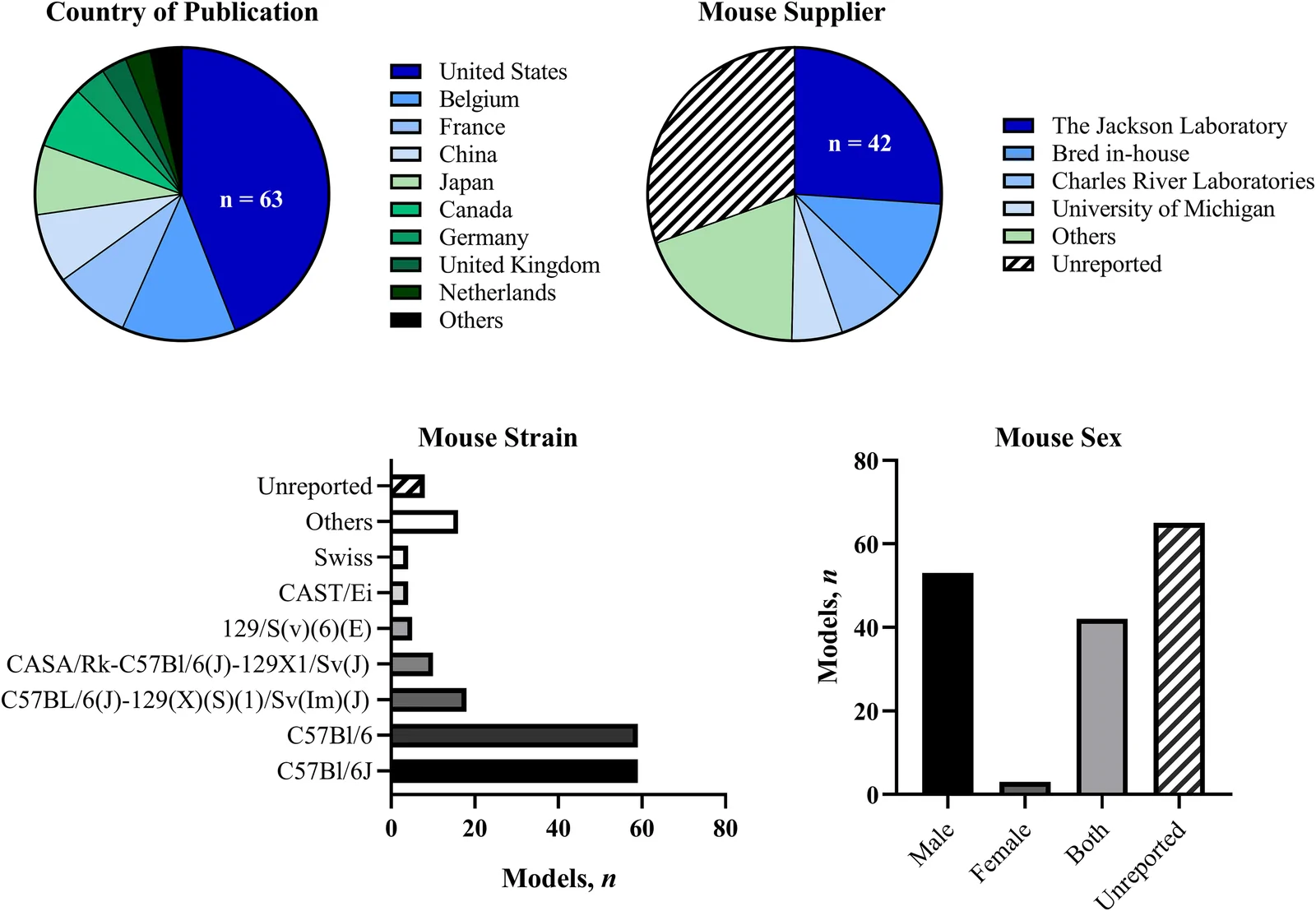
Study characteristics, including country of publication, mouse supplier, mouse strain, and mouse sex reported across studies. Pie charts represent proportions across all included studies, whereas mouse strain and mouse sex bar graphs pertain to all included models, of which there may be more than one per study. Unreported proportions are depicted with hatch marks.

### 3.3 Mouse models of thrombosis involving VWF and/or ADAMTS-13

Mouse models with demonstrated involvement of VWF and/or ADAMTS-13 are detailed in Figure 3 [21,23,[33], [34], [35], [36], [37], [38], [39], [40], [41], [42], [43], [44], [45], [46], [47], [48], [49], [50], [51], [52], [53], [54], [55], [56], [57], [58], [59], [60], [61], [62], [63], [64], [65], [66], [67], [68], [69], [70], [71], [72], [73], [74], [75], [76], [77], [78], [79], [80], [81], [82], [83], [84], [85], [86], [87], [88], [89], [90], [91], [92], [93], [94], [95], [96], [97], [98], [99], [100], [101], [102], [103], [104], [105], [106], [107], [108], [109], [110], [111], [112], [113], [114], [115], [116], [117], [118], [119], [120], [121], [122], [123], [124], [125], [126], [127], [128], [129], [130], [131], [132], [133], [134], [135]]. Details including methodological variations and positive outcomes pertaining to VWF and/or ADAMTS-13 are outlined in Supplementary Table 2. Twenty-nine single-vessel models, 4 organ-specific models, and 12 systemic thrombosis models involving VWF and/or ADAMTS-13 were identified. The most common method of thrombosis induction in a single vessel was ferric chloride [23,[33], [34], [35], [36], [37], [38], [39], [40], [41], [42], [43], [44], [45], [46], [47], [48], [49], [50], [51], [52], [53], [54], [55], [56], [57], [58], [59], [60], [61], [62], [63], [64], [65], [66], [67], [68], [69], [70], [71], [72], [73], [74], [75], [76], [77]], followed by laser injury [39,66,71,[78], [79], [80], [81], [82], [83], [84], [85], [86], [87], [88], [89]], then transient middle cerebral artery occlusion (tMCAo) using a filament [39,46,64,102,[136], [137], [138], [139], [140], [141], [142], [143], [144], [145], [146], [147], [148]]. Most organ-specific models pertained to left anterior descending artery ligation [[106], [107], [108], [109], [110], [111], [112], [113]]. followed by renal artery clamping [7] or ligation [114]. The most common systemic thrombosis model studied was congenital TTP [53,56,98,99,[116], [117], [118], [119], [120], [121], [122], [123], [124], [125], [126], [127]], followed by intravital imaging after administration of an endothelial activating agent (ie, histamine [58,100,101], histones [132], 1-8-deamino-d-arginine vasopressin (DDAVP) [71], or CD40L [133]). Most studies assessed VWF or ADAMTS-13 involvement using knockout (KO) models, as well as therapeutic administration of recombinant ADAMTS-13, anti-VWF antibodies, or fragments of VWF domains that inhibit endogenous VWF binding. Three studies assessed VWF involvement through genetic knockin of fluorescent VWF, showing its integration within the thrombus [33,71,76].

**Figure 3:**
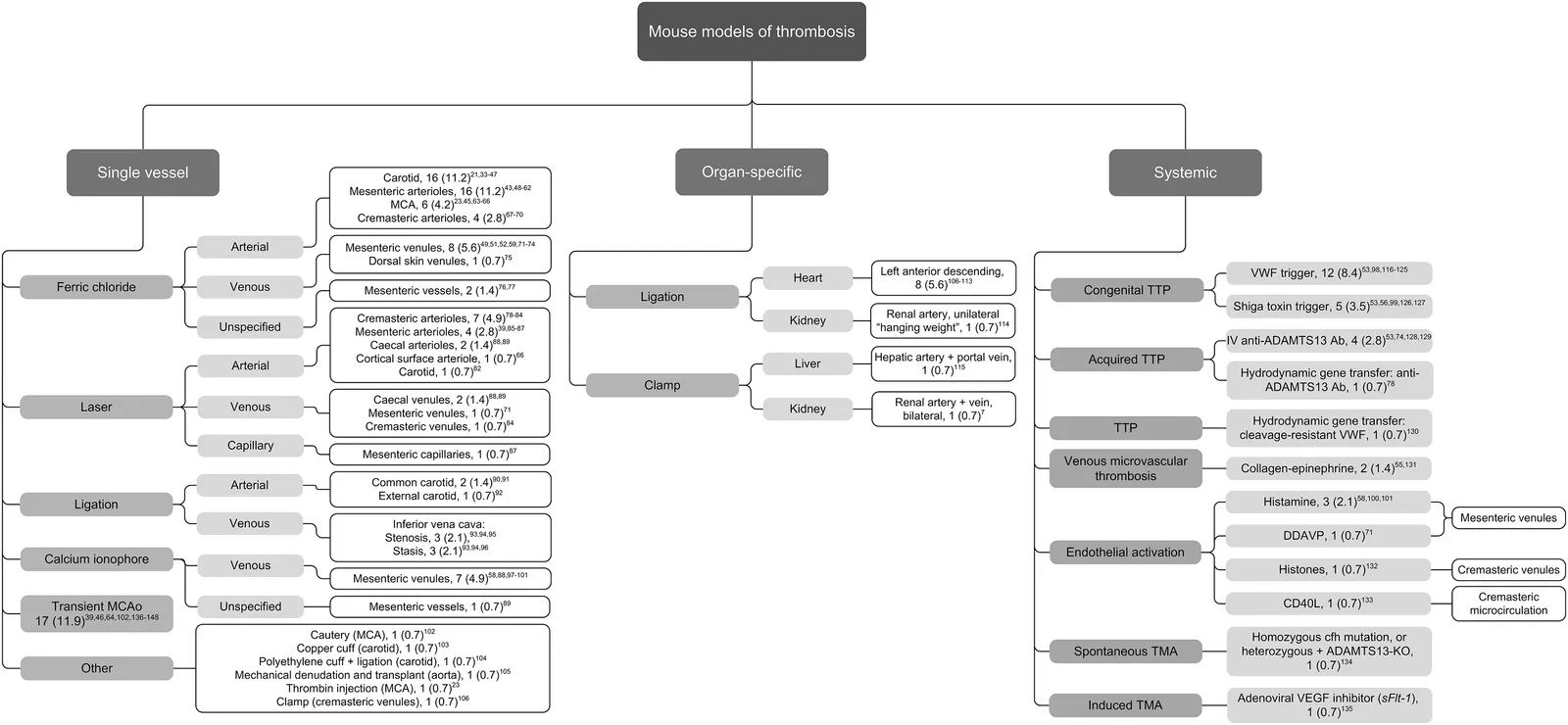
Flow chart outlining mouse models of thrombosis with demonstrated involvement of VWF and/or ADAMTS-13. The number of studies, *n* (%), is indicated next to each model. Ab, antibody; ADAMTS-13, a disintegrin and metalloproteinase with a thrombospondin type 1 motif, member 13; CD40L, cluster of differentiation 40 ligand; DDAVP, 1-8-deamino-d-arginine vasopressin (desmopressin); IV, intravenous; KO, genetic knockout mouse; MCA, middle cerebral artery; MCAo, middle cerebral artery occlusion; sFlt-1, soluble Fms-like tyrosine kinase-1; TTP, thrombotic thrombocytopenic purpura; VEGF, vascular endothelial growth factor; VWF, von Willebrand factor.

There was significant methodological variability among studies that reported the details of their methods (Supplementary Table 2). In ferric chloride-based models, there was variation of ferric chloride concentration, application time, filter paper size, the size of the vessels chosen for imaging, and the length of time the vessel was monitored for occlusion or other thrombotic events. Laser injury models varied by use of photosensitizing agent, type of sensitizing agent and its dosing, light wavelength, as well as the vessel size chosen for injury. Calcium ionophore application models [58,88,89,[97], [98], [99], [100], [101]] varied in concentration of ionophore, duration of observation, and vessel size chosen. tMCAo models varied in the suture size, type, and duration of occlusion. Ligation models of the left anterior descending artery, renal artery, inferior vena cava (IVC) [[93], [94], [95], [96],^149^], and carotid artery [90,91] varied in terms of suture size, suture type, transient vs permanent ligation, and method of endothelial damage when performed (ie, ligating vigorously [90] or crushing with forceps [91]). There were a number of single-vessel models identified that were only cited as involving ADAMTS-13 and/or VWF by a single study (eg, cautery [102], copper cuff [103], polyethylene cuff and ligation [104], mechanical endothelial denudation and transplant [105], and thrombin injection [23]).

Systemic thrombosis model methodologies varied as well. Among congenital TTP models using VWF or Shiga toxin as a trigger, type and dose of the triggering agent and source of VWF varied. Acquired TTP models [53,74,78,128,129] varied by source of VWF, time between anti-ADAMTS-13 antibody and VWF trigger administration, and dosing of both antibodies and the VWF trigger. Collagen-epinephrine injection as a model of venous microvascular thrombosis [55,131] varied by the time between outcome assessment and injection. Finally, systemic endothelial activation was induced by histamine, DDAVP, histones, or CD40L, followed by intravital microscopy of either the mesenteric[58,71,100,101] or cremasteric[132,133] microcirculation. The primary variation found was the route of histamine administration and duration of vessel observation in the histamine model; otherwise, each of the other activation agents was used by only 1 study.

Sixteen disease models with secondary thrombosis outcomes involving VWF and/or ADAMTS-13 were identified (Table 1 [9,84,[150], [151], [152], [153], [154], [155], [156], [157], [158], [159], [160], [161], [162], [163], [164], [165], [166], [167], [168], [169], [170], [171]]), with the most common being sickle cell disease [[150], [151], [152], [153]], followed by atherosclerosis [[154], [155], [156]]. VWF involvement was primarily shown by the presence of VWF within thrombi, vascular surface expression of VWF, or the impact of recombinant ADAMTS-13 administration on various coagulation markers.

**Table 1:** Secondary findings of thrombosis involving VWF and/or ADAMTS-13.

Disease modeled	Studies, *n* (%)	VWF/ADAMTS-13-specific positive findings
Sickle cell disease	4 (2.8)	• VWF-positive hepatic vaso-occlusions, colocalized with RBCs (↓ by **rADAMTS-13**) [150]; ↑ VWF vascular surface expression on aorta [151] • **rADAMTS-13**: ↓ % stasis in dorsal skin venules [152] • **plt-VWF only**: ↑ sickle RBC occlusions, ↑ collagen deposition and ↑ inflammation in liver; ↑ collagen deposition in kidney; ↓ TNF-induced vaso-occlusion in liver, kidneys, lungs, heart, and brain [153]
Atherosclerosis	3 (2.1)	• ↑ VWF/plt adhesion to thoracic aorta (↑ in ***Adamts13*-KO**, reversed by **rADAMTS-13**) [154], ↑ molecular signal on ascending aorta (↓ by **rADAMTS-13**) [155] • ***Vwf-*KO, plt-VWF, or plt-VWF + *Adamts13*-KO under inflammatory conditions**: ↓ plt and leukocyte adherence to carotid [156]
Endotoxemia (LPS administration)	2 (1.4)	• ***Vwf-*KO**: ↑ VWF vascular surface expression in cremaster venules [84] • **rADAMTS-13**: ↓ D-dimer, ↓ hepatic VWF-positive thrombi in liver, ↓ hepatic fibrin deposition [157]
Blood-induced subarachnoid hemorrhage	2 (1.4)	**rADAMTS-13**: ↓ number of large parenchymal vessels with fibrinogen deposition [158]; ↓ fibrinogen staining in cortex (in *Adamts13*-KO) [159]
TBI-induced coagulopathy	2 (1.4)	• **rADAMTS-13**: ↑ clotting time (Century Clot analyzer), ↓ D-dimer [160] • **rVWFA2**: ↑ cerebral blood flow, ↑ fibrinogen, ↓ D-dimer, ↓ fibrin deposition in glomerular microvasculature [161]
*Staphylococcus aureus* sepsis	1 (0.7)	• ***Vwf*-KO:** ↓ hepatic microthrombi • ***Adamts13*-KO**: ↑ renal and hepatic microthrombi • **rADAMTS-13**: ↓ hepatic microthrombi in *Adamts13*-KO [162]
Diabetic nephropathy	1 (0.7)	***Adamts13*-KO**: ↑ glomerular plt staining, ↑ glomerular ECM deposition [163]
AKI (polytrauma and hemorrhagic shock-induced)	1 (0.7)	↑ VWF-positive renal microvascular thrombi [164]
Dehydration/hypernatremia	1 (0.7)	↑ hepatic VWF surface expression [165]
Heart failure (HF)	1 (0.7)	Early and acute decompensated HF have endocardial VWF-positive thrombi; ↑ endocardial sectional % VWF stain in overall HF and early HF [166]
Cerebral cavernous malformations	1 (0.7)	↑ VWF surface expression in cerebellar vessels in lesions, ↑ VWF aggregated in large lesion lumens, with thrombomodulin colocalization [167]
Diet-induced liver steatosis	1 (0.7)	***Adamts13*-KO**: ↓ PAP, ↓ TAT (on SFD), ↑ number of hepatic thrombi, ↑ % of hepatic vessels with VWF-positive occlusive thrombi, ↑ plts in liver (on HFD) [168]
Acute colitis (DSS-induced)	1 (0.7)	***Vwf*-KO**: ↓ endogenous thrombin potential, ↓ peak thrombin [9]
Aging	1 (0.7)	↑ VWF/plt aggregates in brain microvasculature [169]
Ponatinib treatment	1 (0.7)	↑ VWF/plt aggregation on aortic endothelium (↓ by **rADAMTS-13**) [170]
Premature aging (*Bmal1*-KO)	1 (0.7)	↑ VWF-positive penile thrombi in spontaneous priapism [171]

Bolded items in the outcomes column indicate alterations made to the model that led to the listed outcomes relative to control (eg, rADAMTS-13, administration of recombinant ADAMTS-13; rmVWFA1, administration of recombinant mouse VWFA1 fragment; *Vwf*-KO*,* performing model on *Vwf-*KO mice). Outcomes listed without an alteration pertain to the model itself.

ADAMTS-13, a disintegrin and metalloproteinase with a thrombospondin type 1 motif, member 13; AKI, acute kidney injury; DSS, dextran sulfate sodium; ECM*,* extracellular matrix; HFD, high-fat diet; -KO*,* genetic knockout mice; LPS*,* lipopolysaccharide; PAP, plasmin-antiplasmin; plt, platelet; plt-VWF, *Vwf-*KO mice with platelets containing VWF; RBC, red blood cell; SFD, standard fat diet; TAT, thrombin-antithrombin; TBI, traumatic brain injury; TNF, tumor necrosis factor; VWF, von Willebrand factor.

## 4 Discussion

This scoping review is the first study to systematically identify mouse models of thrombosis demonstrating functional involvement of VWF and/or ADAMTS-13, and to compare methodological features across studies that may influence results. Knowledge gaps and recommendations for future research are outlined in Table 2. Considerable heterogeneity was observed in model selection, vessel type, and key methodological features (eg, vessel size, duration of observation, dosing, and injury parameters). Each model carries distinct advantages and limitations that must be weighed carefully [20]. Important biological variables with implications for validity and generalizability, such as mouse supplier, sex, age, weight, and strain, were also frequently underreported. In single-vessel models, vessel type and size are particularly important because shear conditions influence thrombus composition and mechanisms of thrombosis, shaping the clinical relevance of each model [172,173].

**Table 2:** Gaps in knowledge and potential solutions.

Research gap	Description	Implications for future research
Sex as a biological variable	Most studies did not use both male and female mice, and many did not report mouse sex.	Include both male and female mice in studies to account for and compare sex differences in VWF-mediated thrombosis.
Age as a biological variable	Significant variation in the age of mice used across studies, and many did not report mouse age.	Standardize age selection criteria to improve comparability and manipulate to clarify age-related differences in VWF-mediated thrombosis.
Impact of mouse strain on VWF-mediated thrombogenicity	The majority of single-vessel models used C57BL/6 mice, whereas TTP models generally used a mixed CASA/Rk-C57BL/6J-129X1/SvJ strain. It is known that CAST/Ei and CASA/Rk strains have altered VWF biology.	Further exploration of single-vessel thrombosis models using mice with genetic backgrounds of CASA/Rk and/or CAST/Ei is warranted, given that these strains yield improved replication of clinical TTP signs in TTP models when compared with C57BL/6-129/Sv alone.
Heterogeneity in model choice and methodology	A large number of models were used, all with varying methods and choice of arterial or venous circulation.	Standardize thrombosis model methodologies, with inclusion of both venous and arterial assessment when able, to improve comparability of studies and further clarify the role of VWF across the vasculature. Justification for choice of model is warranted given their varying pathogenic mechanisms and clinical applicability.
Thrombus composition	A minority of studies investigated the composition of formed thrombi, with many identifying varying distribution and proportion of VWF and platelets vs fibrin and RBCs.	The clinical importance of thrombus composition (ie, white vs red thrombi) is increasingly apparent, with known differences in response to thrombolysis in mice and humans. Studies should assess thrombus composition within their model for improved clinical translation.
Underreporting of mouse characteristics and detailed methodologies	Mouse characteristics were underreported across studies. Numerous studies were omitted from methodological variation assessment as they did not report detailed methods.	Standardize required reporting of key variables that can influence outcomes, validity, and comparability of studies (ie, mouse age, sex, weight, strain, supplier, and detailed methodology).

RBC*,* red blood cell; TTP*,* thrombotic thrombocytopenic purpura; VWF*,* von Willebrand factor.

Sex was inconsistently reported, and female mice were underrepresented despite known differences in thrombogenicity. Male mice are more vulnerable to thrombosis, partly due to sex-specific regulation of anticoagulant proteins, such as protein C and antithrombin [174]. Estrogen also stimulates VWF synthesis in endothelial cells, enhancing platelet adhesion and clot formation, and shifts coagulation factor and cytokine expression toward a prohemostatic profile [175,176]. Female mice also demonstrate heightened platelet sensitivity to agonists (eg, thrombin and collagen-related peptides), increasing fibrinogen binding and aggregation [177]. These sex-specific differences in thrombogenicity are not unique to mice [178]; failure to account for these differences may obscure mechanistic insights and reduce translational relevance.

Similar concerns apply to the specific mouse strain used, which strongly influences VWF/ADAMTS-13 biology (Table 3 [55,99,127,[179], [180], [181], [182], [183], [184]]). In contrast to humans, complete ADAMTS-13 deficiency alone does not cause TTP in C57BL/6-129/Sv mice or either parental strain [99,127], and backcrossing these mice into the CASA/Rk or CAST/Ei backgrounds is required to model spontaneous or trigger-induced TTP. Backcrossing into CASA/Rk significantly increases plasma VWF levels due to genetic modifiers of VWF synthesis [184] and reduces ultra-large VWF multimers due to expression of the long ADAMTS-13 isoform [55,99,182]. However, higher plasma VWF alone is not a risk factor for mortality, degree of thrombocytopenia, or baseline platelet count [99,127], and one study reported no difference in thrombus formation in ADAMTS-13-deficient mice in C57BL/6 vs CAST/Ei backgrounds [53]. C57BL/6 mice are known to express a truncated form of ADAMTS-13 due to the presence of an IAP-retrotransposon element in intron 23 that leads to an ADAMTS-13 gene product that is truncated at the thrombospondin type 1 repeat number 6 domain. This truncated ADAMTS13 is known to exhibit reduced VWF- multimer-cleaving activity contributing to enhanced thrombotic response in both the ferric chloride injury model and the collagen-epinephrine injury model [56]. Care must therefore be taken when interpreting thrombosis model data generated with C57BL/6 mice to account for this known strain effect. In humans, VWF levels are variable, and patients in clinical remission from TTP may still exhibit ultra-large VWF multimers with decreased ADAMTS-13 activity [10,185]. Moreover, identical ADAMTS-13 mutations can lead to variable disease severity, implying additional modifiers [186]. Although C57BL/6 mice exhibit a milder TTP phenotype, they remain the most common strain used in thrombosis models involving VWF–ADAMTS-13 due to their amenability to genetic manipulation and thrombotic reproducibility.

**Table 3:** Genotype-phenotype traits of mouse strains as it pertains to VWF and ADAMTS-13.

Strain	Traits	Advantages	Disadvantages
C57BL/6	• Insertion of IAP retrotransposon including premature stop codon, resulting in expression of truncated ADAMTS-13 (lacks distal 2 TSP1 repeats and CUB domains) [179] • Moderate-high plasma VWF levels with normal clearance [180]	• Most common inbred strain with extensive genetic tools, knockout libraries, and well-characterized background, suitable for gene targeting studies • Useful to study the role of distal ADAMTS-13 domains • VWF levels provide comparable baseline to humans	• ADAMTS-13 protein does not recapitulate human ADAMTS-13 structure, limiting translational value (activity is ∼10-fold lower) [55,179] • Pure C57BL/6 mice are resistant to spontaneous TTP [99] • Higher tail-clip bleeding assay variability than other strains [181]
129/Sv	• No IAP retrotransposon, expresses ADAMTS-13 with similar domain organization as humans [182] • Moderate plasma VWF levels with normal clearance [180]	• Full-length ADAMTS-13 expression makes this strain ideal for the study of ADAMTS-13 biology • Commonly used for embryonic stem cell-derived gene targeting, making it a natural strain for many knockout models	• Pure 129/Sv mice are resistant to spontaneous TTP [99] • Less extensive reagent and tool availability than C57BL/6 strain
CASA/Rk	• ADAMTS-13 structure and biology not well characterized • Extremely high VWF levels, 10-fold higher than C57BL/6 strain (*Mvwf2*, *Mvwf3*, *Mvwf4* alleles) [183,184]	• Commonly introduced to C57BL/6 and 129/Sv lines to facilitate development of TTP [99] • High VWF phenotype facilitates study of VWF modifier genetics, synthesis, and QTL mapping [184] • *Mvwf3* and *Mvwf4* show conservation of synteny with human chromosomal segments, suggesting translational relevance [184]	• Poorly characterized ADAMTS-13 biology • Wild-derived strain (*Mus musculus castaneus*) with limited genetic tools, reagents, and characterized background • Numerous interacting QTLs create complex genetic architecture • Breeding and colony maintenance may be more challenging
CAST/Ei	• VWF/ADAMTS-13 biology least characterized • VWF levels presumed higher but not explicitly measured [127]	• Can be introduced to C57BL/6 and 129/Sv lines to facilitate development of TTP [99] • High VWF phenotype may be useful for study of VWF modifier genetics, synthesis, and QTL mapping	• Wild-derived strain (*Mus musculus castaneus*) with limited genetic tools, reagents, and characterized background • Largely uncharacterized strain in context of VWF

ADAMTS-13, a disintegrin and metalloproteinase with thrombospondin type 1 motif, member 13; CUB, complement C1r/C1s, Uegf, Bmp1; IAP, intracisternal A-type particle; QTL*,* quantitative trait locus; TSP1, thrombospondin type-1 repeats; TTP*,* thrombotic thrombocytopenic purpura; VWF, von Willebrand factor.

Ferric chloride injury was the most common model of thrombosis. Although ferric chloride is not a physiologic trigger of thrombosis, it causes endothelial cell activation and yields platelet- and VWF-rich thrombi resembling clinically relevant ‘white clots’ [21,65]. These clots are known to be resistant to tPA treatment [27]. However, this may be less applicable to RBC- and fibrin-rich thrombi, or ‘red clots’ [93,95], which are more susceptible to tPA [27]. Methodological variably was substantial. Higher ferric chloride concentrations [45,65], longer exposure [187], and larger filter paper [65] generally produce more stable occlusive thrombi. In contrast, lower concentrations, shorter exposures, and smaller filter papers generate greater variability but may better reflect gradual thrombus formation as seen clinically, with increased sensitivity to therapeutic modulation [188,189]. Vessel size further modifies these effects. Only 3 included studies directly examined how methodological parameters altered VWF-related outcomes (eg, filter paper size [65] and ferric chloride concentrations [36,45]). Although standardization efforts exist [28,29], significant heterogeneity persists.

Laser injury models were similarly heterogeneous. Mechanical laser injury produces focal endothelial disruption, whereas photochemical injury generates reactive oxygen species through activation of intravascular photosensitizers such as Rose Bengal [190]. Compared with ferric chloride injury, the photochemical model may better reflect oxidative mechanisms relevant to arterial thrombosis [191] and COVID-19 [192] and appear to be more sensitive to pharmacologic modulation than mechanical laser injury. Direct comparison shows photochemical thrombi were affected by antiplatelet and anticoagulant agents, whereas mechanically induced thrombi responded only to antiplatelets [190]. Mechanical laser injury, however, offers high spatial precision and better control over injury depth and size [30]. In mechanical models, injury size and depth [85], which are influenced by laser pulse frequency, number of pulses, and energy, affect thrombus size [193], composition [85], and response to therapeutics and genetic perturbations [85,193], yet these parameters were frequently underreported or variable. Only one included study compared models via injury depth and found that *Vwf-*KO mice had variable thrombus formation as a result [85]. In photochemical models, the duration and intensity of excitation can profoundly influence thrombotic outcomes, which varied substantially across studies [194]. Further work is needed to compare VWF-related outcomes across laser injury model types.

Mechanical flow restriction through stasis or stenosis represents another major thrombosis trigger. Given that VWF–ADAMTS-13 interactions are highly shear-dependent, complete stasis may be less physiologically relevant for studying this pathway, although VWF contributes to inflammatory components of stasis and reperfusion [115,139,195] and thrombus formation [94]. The diversity of occlusion methods among included studies introduces numerous opportunities for variability in thrombus formation and pathway activation. Venous models (eg, IVC ligation) employ stasis-driven coagulation pathways, forming RBC- and fibrin-rich thrombi, whereas arterial models (eg, carotid ligation, tMCAo) differ significantly, relying more on platelets and VWF due to higher shear forces. Other key determinants include the degree and duration of occlusion, affecting thrombus composition, size, stability, and immune cell recruitment [173,196]. For example, ADAMTS-13-mediated thrombolysis is more effective than tPA after 4 hours of tMCAo, but similarly effective after 2 hours [148]. Partial IVC ligation models are particularly sensitive to anatomical variation such as side branch location [197]. Suture materials and size can also affect thrombogenicity, inflammation, and reliability of degree of occlusion [[198], [199], [200]]. More rigid devices, including clamps, cuffs, and constrictors [201], generally confer more reproducible thrombosis.

Congenital and acquired TTP models also varied considerably. Congenital TTP was typically induced through *Adamts13* KO or silencing, whereas acquired TTP relied on anti-ADAMTS-13 antibodies administered intravenously or via hydrodynamic gene transfer. In both settings, an additional trigger such as VWF or Shiga toxin was usually required, paralleling the clinical observation that TTP often follows physiological or inflammatory stress [202]. One study used a cleavage-resistant VWF variant (VWF/p.S1494C-p.A1534C) to produce spontaneous TTP without an exogenous trigger, highlighting an alternative approach. However, this method induced massive hepatic injury, complicating interpretation of results [130]. Trigger doses varied widely, strongly influencing disease severity and reproducibility [53]. Given that untreated human TTP has a mortality rate of approximately 90% [202], relatively higher trigger doses may better reflect the natural history of uncontrolled disease [53]. In acquired TTP, the degree of ADAMTS-13 suppression varies with antibody dose and timing of trigger administration [53,74,128,129]. More recent models using patient-derived anti-ADAMTS-13 variable regions [78] are particularly promising as they better reproduce clinically relevant human autoreactivity and may improve translational testing of novel ADAMTS-13 therapeutics [78,203,204].

This review has several limitations. Restricting inclusion to English-language studies may have excluded relevant data. We also focused on studies demonstrating positive functional involvement of VWF and/or ADAMTS-13, excluding work in which these factors were measured but found not to be involved. However, a comprehensive catalog of all negative findings was not feasible within the scope of this review. Studies that assessed VWF by gene expression alone were excluded, limiting insight into transcriptional regulation of VWF but ensuring a focus on data most relevant to clinical translation. Nevertheless, this review highlights substantial methodological heterogeneity and underreporting that limits reproducibility and translational applicability across murine thrombosis studies.

By systematically cataloguing thrombosis models, methodological parameters, and associated VWF- and ADAMTS-13-related outcomes, this review provides a foundation for improving standardization and reporting. The National Preclinical Sepsis Platform successfully implemented cross-institutional standardization of murine sepsis models, improving parameters such as randomization and blinding, standardized protocols and outcomes, consistent timing of therapeutic interventions, uniform endpoints, and inclusion of both sexes [205,206]. This offers a potential template for thrombosis research. Notably, this study aligns with the recommendations of the Stroke Treatment Academic Industry Roundtable, a consortium that seeks to standardize preclinical practices in stroke research to improve the development of new treatments. They also highlight the importance of expanding study populations to include female mice, aged mice, and mice with comorbidities [207]. Greater harmonization of protocols and more comprehensive reporting of biological and technical variables will improve comparability between studies and strengthen the preclinical evaluation of VWF- and ADAMTS-13-targeted therapies.

## 5 Conclusion

This scoping review systemically outlines all mouse models of thrombosis involving VWF and highlights substantial methodological heterogeneity across studies, including differences in mouse sex and strain, vascular bed, thrombosis model, and model-specific protocols. Many of these factors are known to influence thrombotic outcomes, limiting cross-study comparability and the translational strength of preclinical evidence for VWF-targeted therapies. Greater collaboration across research groups is needed to establish standardized, consensus-driven guidelines that improve methodological rigor and reproducibility.
