# Supplementary material for: Mouse models to study von Willebrand factor in thrombosis: a scoping review
Source: Res Pract Thromb Haemost. 2026 Jul 15;10(7):106863. doi: 10.1016/j.rpth.2026.106863 (PMC13675921; doi:10.1016/j.rpth.2026.106863)
Supplement: Supplementary file 1 [file mmc1.docx]

**Supplemental Digital Content 1.** Search terms adapted for MEDLINE:

1. von Willebrand Factor/
2. VWF.mp.
3. von willebrand factor*.mp.
4. ADAMTS13 Protein/
5. ADAMTS13.mp.
6. ADAMTS-13.mp.
7. mice.mp.
8. mouse.mp.
9. exp Mice/
10. or/1-6
11. or/7-9
12. and/10,11
13. limit 12 to English
14. limit 13 to (comment or editorial or letter or meta analysis or “review” or “systematic review”)
15. 13 not 14

**Supplemental Table 1.** Characteristics of all included studies.

| **Author** | **Country** | **Mouse supplier** | **Mouse strain** | **Genetic modifications included** | **Mouse sex** | **Model(s)** | **Age of mice (weeks)** | **Weight of mice (grams)** |
| --- | --- | --- | --- | --- | --- | --- | --- | --- |
| Plautz, 2021[1] | USA | NR | C57BL/6 | WT | M | Trauma-induced AKI | "Adult" | NR |
| de Maat, 2021[2] | Netherlands | NR | CASA/RK-C57BL/6J-129X1/SvJ | *Adamts13*-KO | M and F | Congenital TTP | 8 to 12 | NR |
| Griffin, 2021[3] | USA | NR | C57BL/6 | WT | NR | Ligation (carotid) | NR | NR |
| Shentu, 2021[4] | USA | NR | C57BL/6 | WT, *LdlR-Apobec-1*-KO | NR | Ligation (LAD) | 20 to 25 | NR |
| Cui, 2021[5] | China | Fourth Military Medical Uni. | C57BL/6J | WT | M | TBI-induced coagulopathy | 8 to 12 | 20 to 25 |
| Zhu, 2020[6] | China | NR | C57BL/6 | WT | NR | Ligation (carotid) | 8 to 12 | 18 to 15 |
| Ozawa, 2020[7] | USA | NR | C57BL/6 | WT, *LdlR-*KO, *LdlR-Adamts13-*KO | NR | Atherosclerosis | 20 to 30 (diet began at 14) | NR |
| Denorme, 2021[8] | Belgium | NR | C57BL/6 | WT, *Vwf-*KO | NR | tMCAo | 10 | NR |
| Jin, 2020[9] | China | NR | NR | NR | M | FeCl₃ (carotid) | 6 | 21 to 25 |
| Tahir, 2020[10] | Germany | Charles River, Jackson | C57BL/6 | WT, *Adamts13-*KO | M | Endothelial activation (CD40L) | NR | NR |
| Michels, 2020[11] | Canada | Jackson | C57BL/6J | WT, *Vwf-*KO, *Adamts13-*KO | M and F | IVC stenosis | 6 | Weight-based model |
| Johnston, 2020[12] | USA | NR | C57BL/6 | WT, hPF4/FcyRIIA-double transgenic mice on a *Cxcl4*-KO background (HIT mice) | NR | Laser (carotid, cremasteric art.) | NR | NR |
| Li, 2020[13] | China | NR | NR | NR | NR | IVC stasis | NR | NR |
| Zheng, 2019[14] | USA | Uni. of Michigan | C57BL/6J | WT, *Adamts13*-KO, *Adamts13-*KO + *cfh*W1206R (heterozygous), or *cfh*R1206R (homozygous) | M and F | Spontaneous TMA | 16 days to 3 mos | NR |
| Latifi, 2019[15] | USA | NR | C57BL/6 | WT, *ApoE-*KO | NR | Ponatinib treatment | 15 to 20 (diet began 6 prior) | NR |
| Adili, 2019[16] | USA | Uni. of Michigan | C57BL/6J | WT, *Adamts13-*KO | M and F | Congenital TTP, calcium ionophore (mesenteric ven.) | 8 to 10 (TTP), 4 (calcium ionophore) | NR |
| Nimjee, 2019[17] | USA | Jackson | C57BL/6 | WT | M and F | FeCl₃ (carotid) | 8 | NR |
| Brait, 2019[18] | Spain | NR | C57BL/6, BALB/c | WT, *Cd69*-KO | M | tMCAo | 3 to 4 mos | NR |
| Slobodianuk, 2019[19] | USA | NR | C57BL/6J | WT, *Vwf-*KO, VWF variant R1399H (hetero-, homo-, and hemizygous) knockin | M and F | FeCl₃ (carotid) | 7 to 14 | NR |
| South, 2018[20] | UK | NR | C57BL/6 | WT | NR | FeCl₃ (MCA) | 6 to 8 | NR |
| Wan, 2018[21] | Canada | Charles River | C57BL/6 | WT, *Vwf-*KO, *Adamts13-*KO | M and F | Subarachnoid hemorrhage | 90 days | 18 to 30 |
| Ozawa, 2018[22] | USA | NR | C57BL/6 | WT, *Adamts13-*KO | NR | Ligation (LAD) | NR | 23.8 to 24.2 |
| Abdelgawwad, 2018[23] | USA | NR | NR | WT, *Adamts13-*KO | NR | FeCl₃ (mesenteric art.) | 4 | NR |
| Kanaji, 2018[24] | USA | NR | C57BL/6J | WT, combinations of humanized GPIbα knock-in, humanized VWFA1 and/or A2 domain knock-in, *Vwf-*KO, and *GpIbα*-KO | NR | FeCl₃ (carotid) | NR | NR |
| Moccetti, 2018[25] | USA | NR | C57BL/6 | WT, *LdlR-Apobec-1*-KO | NR | Ligation (LAD) | 20 to 25 | NR |
| Nicolay, 2018[26] | Germany | Jackson, Charles River | C57BL/6J, C57BL/6 | WT, *Vwf-*KO | M | Ligation (renal artery) | 4 to 5 mos | NR |
| Urisono, 2018[27] | Japan | Jackson, Japan SLC | C57BL/6 | WT, *Vwf-*KO, CAG-EGFP, or *Vwf*-KO + CAG-EGFP | M | Clamp (hepatic artery & portal vein) | 8 to 12 | NR |
| Doddapattar, 2018[28] | USA | NR | C57BL/6J | *ApoE-*KO, *ApoE*-*Vwf-*KO | F | Atherosclerosis | 10 to 11 | NR |
| Geys, 2018[29] | Belgium | NR | CASA/RK-C57BL/6J-129X1/Sv | WT, *Adamts13-*KO | M | Congenital TTP | 20 (diet began at 5) | Weight-based model |
| Jackel, 2017[30] | Germany | LFB Biomedicament | C57BL/6J | WT, *Tlr2*-KO, *Vwf*-KO | M and F | Ligation (carotid) | 8 to 14 | 20+ |
| Tersteeg, 2017-1[31] | Belgium | NR | CASA/RK-C57BL/6J-129X1/SvJ | *Adamts13*-KO | NR | Congenital TTP | NR | NR |
| Tersteeg, 2017-2[32] | Belgium | Jackson | C57BL/6, C57BL/6-129/SvJ | C57BL/6: WT, C57BL/6-129/SvJ: WT, *α2-antiplasmin*-KO | M | Acquired TTP, FeCl₃ (mesenteric ven.) | NR | 16 to 25 |
| Aymé, 2017[33] | France | NR | C57BL/6J | WT | NR | FeCl₃ (mesenteric art. & ven.) | 3 to 4 | NR |
| Swystun, 2017[34] | Canada | NR | C57BL/6 | *FVIII*-KO, *Vwf-*KO or *Vwf-FVIII*-KO with hydrodynamic gene transfer of WT VWF, variant (R816W, R763A, R854Q, R816W/R854Q) | NR | Laser (cremasteric art.) | NR | NR |
| Dhanesha, 2017[35] | Japan | NR | C57BL/6J | WT, *Adamts13-*KO, *Adamts13*-*Vwf-*KO | M | Diabetic nephropathy | 6 to 8 | NR |
| Han, 2017[36] | China | Jackson, Vital River Ltd. Co. | C57BL/6J | WT, *Zyxin-*KO | M and F | FeCl₃ (mesenteric vessels) | 8 to 10 | NR |
| Verhenne, 2017[37] | Belgium | In-house, Uni. of Michigan | CASA/RK-C57BL/6J-129X1/SvJ | WT, *Adamts13-*KO | NR | Congenital TTP | 5 to 7 | 16 to 21 |
| Prakash, 2017[38] | USA | NR | C57BL/6J | WT, *Vwf-*KO, *Tsp1*-KO, *Vwf-Tsp1*-KO | NR | Laser (mesenteric art.) | 4 to 5 | NR |
| Geys, 2017[39] | Belgium | NR | CASA/RK-C57BL/6J-129X1/Sv | WT, *Adamts13*-KO | M | Diet-induced liver steatosis | 5+ | Weight-based model |
| Kim, 2017[40] | South Korea | Jackson, Orient | C57BL/6 | WT, *Adamts13*-KO | M | Clamp (renal artery & vein) | 6 to 8 | 20 to 25 |
| Kristofik, 2016[41] | USA | NR | C57BL/6-129/SvJ | WT, *Tsp2*-KO | NR | Mechanical denudation and transplant (aorta) | 8 to 12 | NR |
| Denorme, 2016[42] | Belgium | Jackson | CASA/RK-C57BL/6J-129X1/SvJ, C57BL/6J | C57Bl/6J-CASA/RK-129X1/SvJ: WT, *Adamts13*-KO; C57Bl/6J: WT | M and F | FeCl₃ (MCA) | 8 to 12 | NR |
| Chung, 2016[43] | USA | NR | C57BL/6 | *Adamts13*-KO | NR | Congenital TTP | 14 | NR |
| Le Besnerais, 2016[44] | France | NR | C57BL/6-129X1/Sv | WT, *Adamts13*-KO | M and F | Congenital TTP | NR | 19 to 25 |
| Deforche, 2016[45] | Belgium | In-house | CASA/RK-C57BL/6J-129X1/SvJ | WT, *Adamts13*-KO | M and F | Acquired TTP | 8 | NR |
| Dhanesha, 2016[46] | USA | In-house | C57BL/6J | WT, *Adamts13*-KO, *Vwf*-KO, *Adamts13*-*Vwf*-KO | M (FeCl₃, tMCAo), M and F (laser) | FeCl₃ (carotid), laser (mesenteric art.), tMCAo | 8 to 10 (FeCl₃, tMCAo), 4 to 5 (laser) | NR |
| Kopic, 2016[47] | Austria | NR | C57BL/6J-129S1/SvImJ | *Adamts13*-KO | M and F | Congenital TTP | NR | NR |
| Adam, 2016[48] | France | genOway | C57BL/6 | WT, knockin of VWF variant V1316M (hetero- and homozygous) | NR | FeCl₃ (mesenteric ven. & art.) | 4 to 5 | NR |
| Fan, 2016[49] | China | Shanghai SLAC | C57BL/6J | WT, *Adamts13*-KO | M | tMCAo | "Adult" | 23 to 26 |
| Ostertag, 2016[50] | USA | Jackson | C57BL/6 (laser), CAST/Ei (TTP) | Hydrodynamic gene transfer of human anti-ADAMTS13 scFv’s | NR | Acquired TTP, laser (cremaster art.) | 2 to 3 mos | NR |
| Erpenbeck, 2016[51] | USA | NR | C57BL/6J | WT, *Adamts13*-KO, adenoviral gene transfer of *sFlt-1* | NR | Induced TMA | 11 to 12 | NR |
| Verhenne, 2015[52] | Belgium | NR | C57BL/6J | WT, *Vwf*-KO | NR | FeCl₃ (carotid), tMCAo | 11 to 14 | NR |
| Pickens, 2015[53] | USA | In-house, Uni. of Michigan | CAST/Ei (TTP, FeCl₃), C57BL/6 (FeCl₃) | WT, *Adamts13*-KO, knockin of platelets expressing ADAMTS13 | NR | Congenital TTP, FeCl₃ (mesenteric art.) | 4 to 8 (TTP), 4 (FeCl₃) | NR |
| Prakash, 2015[54] | USA | Jackson | C57BL/6J | WT, *Vwf*-KO, *Tsp1*-KO, *Vwf-Tsp1*-KO, *Tsp1-*KO + *Vwf*^+/-^ | M and F | FeCl₃ (mesenteric art.) | 4 to 5 | 13 to 15 |
| Nakano, 2015[55] | Japan | Kiwa Experimental Animal Laboratory | ddY | WT | M | tMCAo | 6 to 8 | 25 to 35 |
| Shim, 2015[56] | USA | NR | C57BL/6 | *LdlR-Apobec-1*-KO | NR | Atherosclerosis | 10, 20, 30, 40 | NR |
| Li, 2015[57] | China | Anhui Medical Uni. | Kunming | WT | M | tMCAo | 6 to 8 | 26 to 28 |
| De Cock, 2015[58] | Belgium | Baxter Innovations GmbH | NR | WT, *Adamts13*-KO, hydrodynamic gene transfer of ADAMTS13 mutant D187H | NR | Congenital TTP | 4 to 8 | 15 to 20 |
| Chen, 2014[59] | USA | Jackson | C57BL/6J | WT, *Adamts13*-KO | NR | Laser (cremasteric art.) | 8 to 12 | NR |
| Shida, 2014[60] | Canada | Queen's Uni. | C57BL/6 | *Vwf*-KO, hydrodynamic gene transfer of VWF mutants S1731T, W1745C, L1757A | NR | FeCl₃ (cremasteric art.) | 10 to 12 | NR |
| Xu, 2021[61] | USA | Jackson | C57BL/6J | WT, *Adamts13*-KO | M | TBI-induced coagulopathy | 12 to 16 | 22 to 25 |
| Bao, 2014[62] | USA | NR | NR | WT, *Adamts13*-KO | NR | FeCl₃ (mesenteric ven.) | NR | 12 to 15 |
| Morioka, 2014[63] | Japan | NR | C57BL/6 | WT, *Vwf*-KO, hydrodynamic gene transfer of VWF mutants S1494C/S1534C, S1494C, S1534C | NR | TTP | NR | NR |
| Le Behot, 2014[64] | France | CURB | C57BL/6, Swiss | WT, *Vwf*-KO | M | FeCl₃ (MCA, carotid) | 15 to 18 | 35 ± 2 |
| Vergouwen, 2014[65] | Netherlands | Charles River | C57BL/6J | WT, *Adamts13*-KO | M | Subarachnoid hemorrhage | NR | 22 to 30 |
| Legendre, 2013[66] | France | NR | C57BL/6 | *Vwf*-KO, hydrodynamic gene transfer of WT VWF, VWF mutants L1696R, P1824H (hetero- or homozygous) | NR | FeCl₃ (mesenteric art. & ven.) | NR | NR |
| Wang, 2013[67] | China | Shanghai SLAC | C57BL/6J | WT | M | tMCAo | 10 | 23 to 26 |
| Zheng, 2016[68] | USA | Uni. of Michigan | CAST/Ei | *Adamts13*-KO | NR | Congenital TTP | NR | NR |
| Gandhi, 2012[69] | USA | Jackson | C57BL/6J, C57BL/6 | C57BL/6J: WT, C57BL/6: *Vwf*-KO, *Adamts13*-KO, *Vwf*-*Adamts13*-KO | M | Ligation (LAD) | 8 to 10 | NR |
| De Meyer, 2012[70] | USA | Jackson | C57BL/6J | WT, *Adamts13*-KO | M | Ligation (LAD) | 8 to 10 | NR |
| Crescente, 2012[71] | USA | Jackson | C57BL/6J | WT | M | FeCl₃ (dorsal skin chamber) | 8 to 10 | NR |
| Khan, 2012[72] | USA | Jackson | C57BL/6J, C57BL/6 | C57BL/6J: WT, heterozygous knockout; C57BL/6: *Vwf*-KO, *Adamts13*-KO, *Vwf-Adamts13*-KO | M | tMCAo | 8 to 10 | NR |
| Pruss, 2012[73] | Canada | Jackson | C57BL/6 | WT, *Vwf*-KO, hydrodynamic gene transfer of VWF mutants R1597W, Y1605A/M1606A, WT ADAMTS13 | M and F | FeCl₃ (cremasteric art.) | 8 to 10 | NR |
| Fujioka, 2012[74] | Japan | In-house | 129/Sv | WT, *Adamts13*-KO | M | tMCAo | 8 to 10 | 20 to 23 |
| Nishimura, 2012[75] | Japan | Charles River, Uni. Hospital Basel | C57BL/6J | WT, knockin of integrin mutant (L746A, Y747A), *Tnfα-*KO,  *Tnfα–R1*-KO, *Il1α/β-*KO,  *Il1RA*-KO, *Pf4-Rac1-*KO | M | Laser (mesenteric art. & capillaries) | 12 | NR |
| Navarrete, 2012[76] | France | Inserm U770 | C57BL/6 | WT, *Vwf*-KO | M and F | FeCl₃ (mesenteric art.) | 4 to 8 | NR |
| Schiviz, 2012[77] | Austria | NR | C57BL/6J-129S1/SvImJ | WT, *Adamts13*-KO | M and F | Congenital TTP | 12 to 18 | NR |
| Xiao, 2011[78] | USA | NR | C57BL/6J | WT, *Adamts13*-KO | NR | FeCl₃ (carotid, mesenteric art.) | 2 to 4 mos | NR |
| Rybaltowski, 2011[79] | Japan | Osaka University | C57BL/6J, C57BL/6 | GFP-expressing WT, GFP-*Adamts13*-KO, *Adamts13*-KO | NR | FeCl₃ (mesenteric ven.), laser (mesenteric ven.) | NR | NR |
| Pruss, 2011[80] | Canada | Jackson | C57BL/6 | WT, *Vwf*-KO, hydrodynamic gene transfer of WT VWF, VWF mutants R1205H, Y1584C (hetero- and homozygous), WT ADAMTS13 | M | FeCl₃ (cremasteric art.) | 8 to 10 | NR |
| Chen, 2011[81] | USA | Jackson, Uni. of Iowa, Uni. of Michigan | C57BL/6 | WT, *Adamts13*-KO | M | Calcium ionophore (mesenteric ven.) | 4 | NR |
| Brill, 2011[82] | USA | Jackson | C57BL/6J | WT, *Vwf*-KO, *Vwf^+/-^,* | M | IVC stenosis | 8 to 10 | NR |
| Huang, 2010[83] | USA | Jackson | C57BL/6J -CAST/Ei | WT, *Adamts13*-KO, *Adamts13^+/-^* | NR | Congenital TTP | NR | NR |
| Golder, 2010[84] | Canada | NR | C57BL/6 | WT, *Vwf*-KO, hydrodynamic gene transfer of WT VWF, VWF mutants R1306W, V1316M, R1341Q | M | FeCl₃ (cremasteric art.) | 8 to 10 | NR |
| Rayes, 2010[85] | France | Uni. of Iowa | C57BL/6 | *Vwf*-KO, *Vwf-Adamts13*-KO | NR | FeCl₃ (mesenteric ven.) | NR | NR |
| Fujioka, 2010[86] | Japan | In-house | NR | WT, *Adamts13*-KO | M | tMCAo | 8 to 10 | 20 to 23 |
| Hemmeryckx, 2011[87] | Belgium | Uni. of Kentucky | C57BL/6J | WT, *Bmal1*-KO | M | Premature aging | 10 to 30 | 17 to 27 (10 wks), 17.3 to 33 (30 wks) |
| Eckly, 2011[88] | France | Charles River, Inserm S770 | C57BL/6J | WT, *Vwf*-KO, *α_IIB_-*KO, *β1*-KO | M | FeCl₃ (carotid, mesenteric art.) | 8 to 10 | NR |
| De Meyer, 2010[89] | Belgium | NR | C57BL/6 | WT, *Vwf*-KO, hydrodynamic gene transfer of WT VWF, VWF variants D1742A/S1783A/H1786A, K1362A, D2509G | M and F | tMCAo | 8 to 10 | NR |
| Hechler, 2010[90] | France | Inserm U770 | C57BL/6 | WT, *Vwf*-KO | M | Laser (mesenteric art.) | 3 to 4 | 15 |
| Dieude, 2009[91] | Canada | Harlan Sprague Dawley | BALB/c | WT | F | FeCl₃ (carotid) | 10 to 12 | NR |
| Guerrero, 2009[92] | USA | In-house | C57BL/6J | WT, knockin of GPIbα variant G233V | NR | FeCl₃ (carotid) | NR | NR |
| Zhao, 2009[93] | USA | Jackson | C57BL/6J | WT, *Vwf*-KO, *Adamts13*-KO, VWF-*Adamts13*-KO, *Vwf^+/-^* | M | tMCAo | 8 to 10 | NR |
| Laje, 2009[94] | USA | Uni. of Michigan | C57BL/6-129/Sv | WT, *Adamts13*-KO | NR | FeCl₃ (carotid) | 5 mos | NR |
| Banno, 2009[95] | Japan | Japan SLC, Clea Japan | C57BL/6-129/Sv, 129/Sv | WT | M and F | FeCl₃ (mesenteric art.) | NR | NR |
| Chauhan, 2008[96] | USA | Jackson | CASA/RK-C57BL/6-129X1/SvJ (TTP), C57BL/6 (FeCl₃) | WT, *Adamts13*-KO, *Vwf*-KO, *Adamts13*-KO *+ Vwf^+/-^,* *Adamts13^+/-^* + *Vwf^+/-^* | M and F | Congenital TTP, FeCl₃ (mesenteric art.) | 6 to 8 (TTP), 3 to 4 (FeCl₃) | NR |
| Kleinschnitz, 2009[97] | Germany | NR | C57BL/6 | WT, *Vwf*-KO | NR | tMCAo | 6 to 8 | NR |
| Marx, 2008[98] | France | NR | C57BL/6 | WT, *Vwf*-KO | M and F | FeCl₃ (mesenteric art.) | 3 to 4 | NR |
| Niiya, 2009[99] | USA | Taconic | C57BL/6J-129/Sv | WT, *Adamts13*-KO, lentiviral gene transfer of ADAMTS13, MDTCS | NR | FeCl₃ (carotid) | 24 | NR |
| Patel, 2008[100] | USA | Jackson | C57BL/6J | WT, *Vwf*-KO, *P-selectin*-KO | M | Laser (cremasteric ven.) | NR | ~30 |
| De Meyer, 2008[101] | Belgium | Jackson | C57BL/6 | WT, *Vwf*-KO, hydrodynamic gene transfer of WT VWF | NR | FeCl₃ (mesenteric vessels) | 4 to 8 | 15 to 25 |
| Chen, 2008[102] | USA | In-house | C57BL/6 | WT, knockin of VWF variant R1326H | M and F | Laser (cremasteric art.) | NR | NR |
| Chauhan, 2007-1[103] | USA | Jackson | C57BL/6J | WT, *Vwf*-KO, *FVIII*-KO, *Vwf^+^*^/-^, knockin of human IL4Rα in place of GPIBα | M and F | FeCl₃ (mesenteric ven.) | 4 | 13 to 17 |
| Banno, 2006[104] | Japan | In-house | C57BL/6-129/Sv | WT, *Adamts13*-KO | F | Collagen-epinephrine | 15 to 20 | 20 to 30 |
| Bonnefoy, 2006[105] | Belgium | NR | Swiss | WT, *Tsp1*-KO | NR | Laser (caecal art. & ven.), calcium ionophore (mesenteric ven.) | 10 to 16 (laser), 4 to 5 (calcium ionophore) | NR |
| Dubois, 2007[106] | USA | Jackson | C57BL/6J | WT, *Vwf*-KO, *FcRγ-*KO | NR | Laser (cremasteric art.) | NR | NR |
| Chauhan, 2007-2[107] | USA | Jackson | C57BL/6J, BALB/c | C57BL/6: WT, *P-selectin*-KO; BALB/c: *β_3_*-KO | M and F | Endothelial activation (histamine) | 4 | 15 to 19 |
| Chauhan, 2006[108] | USA | Jackson, in-house | C57BL/6J-129/Sv, C57BL/6J | WT, *Adamts13*-KO | M and F | FeCl₃ (mesenteric art.), calcium ionophore (mesenteric ven.), endothelial activation (histamine) | 4 | 14 to 18 |
| Motto, 2005[109] | USA | Jackson, in-house | C57BL/6J-129/SvJ, CASA/RK-C57BL/6J-129X1/SvJ | WT, *Adamts13*-KO | M and F | Congenital TTP, calcium ionophore (mesenteric ven.) | 4 to 8 | 14 to 21 |
| Sha, 2005[110] | USA | In-house | C57BL/6 | WT, *Plasminogen*-KO, knockin of an apo(a) 6 KIV construct with liver-specific promotor, *Plasminogen*-KO + apo(a) construct as above | NR | Copper cuff (carotid) | 7 to 9 | NR |
| Sasaki, 2004[111] | Japan | Japan SLC | C57BL/6J | WT | NR | Polyethylene cuff and ligation (carotid) | 8 to 10 | 22 to 26 |
| Andre, 2000[112] | USA | NR | C57BL/6J-129/Sv | WT, *Vwf*-KO, *P-selectin-*KO, β_3_-KO | M | Calcium ionophore (mesenteric ven.) | "Young" | 14 to 19 |
| Ni, 2000[113] | USA | In-house | C57BL/6J-129/Sv | WT, *Vwf*-KO, *Fibrinogen-*KO, *Vwf-Fibrinogen*-KO | M | FeCl₃ (mesenteric art.) | 3.5 to 4.5 | NR |
| Oury, 2006[114] | Belgium | NR | FVB | WT, platelet-specific overexpression of human P2X_1_ | NR | Laser (mesenteric art. and ven.) | 8 to 12 | NR |
| Receveur, 2020[115] | France | Charles River | C57BL/6 | WT, *Vwf*-KO | NR | FeCl₃ (carotid) | 8 to 10 | NR |
| De Maeyer, 2010[116] | Belgium | Uni. of Michigan, in-house | CASA/RK-C57BL/6J-129X1/Sv | WT, *Adamts13*-KO | NR | FeCl₃ (mesenteric art. and ven.) | NR | 12 to 15 |
| Jiang, 2024[117] | China | Shulaibao Biotechnology | 129/Sv | WT, *Adamts13*-KO | M | tMCAo | 6 to 8 | 21 to 23 |
| South, 2024[118] | UK | NR | C57BL/6J | WT | NR | FeCl₃ (MCA), laser (cortical surface art.) | NR | 20 to 25 |
| Bourne, 2024[119] | UK | Harlan Laboratories | C57BL/6 | WT | M and F | FeCl₃ (carotid) | 8 to 12 | NR |
| Schellenberg, 2024[120] | France | Charles River | C57BL/6 | WT, *Vwf*-KO | M | Acute colitis | 8 to 10 | NR |
| Ozawa, 2024[121] | USA | NR | NR | *LdlR-Apobec-1*-KO | M and F | Ligation (LAD) | 20 to 25 | NR |
| Shi, 2024[122] | USA | Jackson | Townes | Knockin of human sickle hemoglobin (β^S^/β^S^) or human WT hemoglobin (β^A^/β^A^) | NR | Sickle cell disease | 1.5 mos, 2 mos | NR |
| Alavi, 2023[123] | Canada | Charles River | C57BL/6NCrl | WT | M | Aging | 3 mos, 18 to 19 mos | NR |
| Gao, 2023[124] | USA | Jackson | C57BL/6J | WT | M and F | Endotoxemia | 10.5 (average) | 19 to 26 |
| Chung, 2023[125] | USA | In-house | C57BL/6 | WT, *Adamts13*-KO, *Adamts13-LdlR*-KO | NR | Congenital TTP | 17 to 19 | NR |
| Ozawa, 2023[126] | USA | Jackson | C57BL/6 | WT, *LdlC-Apobec-1-*KO | M and F | Ligation (LAD), clamp (cremasteric ven.) | 10 to 20 | NR |
| An, 2023[127] | USA | In-house | Townes-129/B6 | Knockin of human α and ^A^γβ^S^ globins in place of murine α- and β-globins | M and F | Sickle cell disease | 11 to 14 | NR |
| Choi, 2023[128] | Canada | Jackson | C57BL/6 | WT | M | IVC stenosis | 8 to 10 | NR |
| De Wilde, 2022[129] | Belgium | NR | C57BL/6J | WT, *Vwf*-KO | M and F | tMCAo | 8 to 12 | NR |
| Shi, 2022[130] | USA | Jackson, Uni. of Michigan, Uni. of Kansas | Townes | Knockin of human sickle hemoglobin (β^S^/β^S^) or human WT hemoglobin (β^A^/β^A^) | NR | Sickle cell disease | NR | NR |
| van Moorsel, 2022[131] | Netherlands | Charles River | OF1 | WT | M | FeCl₃ (MCA) | 12 | NR |
| Globisch, 2022[132] | Sweden | In-house | NR | WT, *Cdh5(PAC)-*Cre-ER^T2^/Ccm3^flox/flox^ | NR | Cerebral cavernous malformations | Postnatal days 1, 6, 7, 8, and 9 | NR |
| Courson, 2022[133] | USA | Jackson | C57BL/6-129 | WT, *Vwf*-KO | M | Endothelial activation (histones) | 12 to 20 | ~30 |
| Stivala, 2922[134] | Switzerland | Jackson | Berkeley | Knockin of human LCR-α1^G^γ^A^γδβ^S^ in place of murine α- and β-globins | M | Sickle cell disease | 8 | NR |
| Xu, 2022[135] | China | Biocytogen | C57BL/6J | WT, GFP-VWF knock-in | M and F | FeCl₃ (carotid) | 6 to 8 | NR |
| Shao, 2022[136] | USA | Jackson | C57BL/6J | WT, platelet *Clec-2*-KO | M and F | Acquired TTP | 16 | NR |
| South, 2022[137] | UK | Jackson | CD1 | WT | M | tMCAo, FeCl₃ (MCA) | 10 to 13 | 25 to 30 |
| Heger, 2024[138] | USA | Jackson | C57BL/6J | WT, *Nlrp3*-KO | M | Ligation (LAD) | NR | NR |
| Sun, 2024[139] | China | NR | C57BL/6 | WT | M | IVC stasis | NR | 18 to 22 |
| Schoner, 2015[140] | USA | Charles River | CD1 | Knockin of dominant negative CREB variant S-A133 | NR | Heart failure | 10 to 24 | NR |
| Dmitrieva, 2014[141] | USA | Taconic | 129/S6, 129/SVE | WT | NR | Dehydration/hypernatremia | 3 mos | ~25 to 26 |
| Jongejan, 2024[142] | Netherlands | Jackson, Charles River | C57BL/6J, C57BL/6J-129S1/SvImJ | WT, WT with allele-selective siRNA silencing of *Vwf*, *Vwf*-KO | M and F | FeCl₃ (mesenteric art. and ven.) | 3 to 4 | NR |
| Peetermans, 2020[143] | Belgium | In-house | C57BL/6 | WT, *Vwf*-KO, *Adamts13*-KO | NR | *Staphylococcus aureus* sepsis | 5 to 11 | NR |

*NR*, not reported; *Uni,* university; *mos,* months; *wks,* weeks; *WT,* wild-type; -*KO,* knockout; *HIT,* heparin-induced thrombocytopenia; *VWF*, von Willebrand Factor; *art,* arterioles; *ven*, venules; FeCl*₃*, ferric chloride; *tMCAo,* transient middle cerebral artery occlusion; *TTP*, thrombotic thrombocytopenic purpura; *MCA*, middle cerebral artery; *TMA*, thrombotic microangiopathy; *LAD*, left anterior descending artery; *IVC*, inferior vena cava; *TBI,* traumatic brain injury; *scFv’s,* single-chain variable-region antibody fragments; *GFP*, green fluorescent protein.

**Supplemental Table 2.** Models of thrombosis with findings indicating VWF and/or ADAMTS13 involvement.

| **Thrombosis type** | **Method of induction** | **Vessel type** | **Vessel site** | **Studies**  **n (%)** | **Key methodological variations** | **VWF/ADAMTS13-specific positive findings** |
| --- | --- | --- | --- | --- | --- | --- |
| **Single vessel** | Ferric chloride | Arterial | Carotid artery | 16 (11.2) | - **Ferric chloride concentration**: 3%,[135] 4%,[115] 6%,[91] 7.5%,[88] 9%,[24] 10%,[17, 19, 46, 64, 78, 92, 94, 99, 119] 12%,[52] 20%[9] - **Filter paper size**: 0.5 x 1 mm,[91] 1 x 1 mm,[52] 1 x 2 mm,[19, 46, 78, 99] 3 x 1 mm,[88] 4 x 10 mm,[92] 2 mm[94] - **Application time**: 1 min,[94, 99] 2 min,[78, 88] 2.5 min,[115] 3 min,[17, 19, 24, 46, 52, 64, 91, 92, 119] 5 min,[135] 15 min[9] - **Monitoring**: 16 min,[88] 1000s,[115] 23 min,[91] 25 min,[119] 30 min,[19, 78, 94, 99, 135] 33 min,[24] 40 min,[46, 92] 50 min,[52] 60 min[17] - Filter paper wrapped around artery[9] - Performed model with or without carotid stenosis[115] | - Fluorescent VWF in thrombus,[135] ↑ VWF vascular surface expression,[91] ↑ VWF/plt stain in thrombus,[119] ↑ plasma VWF and ↓ plasma ADAMTS13 in combination with stress restraint[9] - ***Vwf*-KO**: ↑ time to occlusion,[19, 46, 52] ↑ time to 100 µm thrombus,[46] ↑ blood flow,[52] ↓ thrombus size[88] (compared to plt-VWF[46]), protected from ↑ thrombogenesis with stenosis[115] - **plt-VWF only, or plt-VWF with *Adamts13*-KO**: ↑ time to occlusion[46] (plt-VWF only[52]), ↑ blood flow (plt-VWF only[52]), ↑ time to 100 µm thrombus, ↓ rate of thrombus growth, ↓ thrombus size[46] - ***Adamts13*-KO**: ↓ time to occlusion[94, 99] - **rADAMTS13**: ↑ time to complete occlusion[78] - **VWF aptamer**: ↑ vessel patency, ↑ recanalization[17] - **anti-VWFA1:** ↑ blood flow[64] - **NMC-4 (blocks VWF:GPIbɑ binding)**: ↑ time to occlusion, ↑ time to stable occlusion, ↑ flow index[24] - **Expression of Plt-VWD mutant (G233V)**: ↑ blood flow[92] - **Expression of VWD mutant with reduced collagen binding (1399H)**: ↑ time to occlusion[19] - **Expression of MDTCS**: ↑ time to occlusion, ↓ % occlusion at 30 min[99] |
|  |  |  | Mesenteric arteriole(s) | 16 (11.2) | - **Ferric chloride concentration**: 7.5%,[76, 85] 10%[23, 33, 48, 53, 54, 78, 95, 96, 98, 108, 116, 142] - **Filter paper size**: 0.5 x 4 mm[76, 85, 98] - **Application time:** 2 min,[116] 3 min,[23, 53] 5 min,[54, 76, 85, 95, 96, 98, 108] 6 min[48] - **Monitoring**: 5 min,[116] 30 min,[23, 53, 78] 40 min,[54, 76, 85, 95, 96, 108, 113, 142] 45 min,[98, 142] 60 min[33, 48] - **Vessel size**: 80-120 µm,[113] 90-130 µm,[76] 90-120 µm,[78] 100-130 µm,[85, 98] 100-150 µm,[54, 95] 100 µm,[96, 108] 109 ± 5 µm[116] - Applied ferric chloride topically[33, 48] | - ***Vwf*-KO**: ↓ plt deposition,[54] ↓ plt deposition 3-5 min post-injury,[113] ↓ plt string formation,[116] ↓ thrombus growth rate,[54] ↑ time to occlusion,[54, 96, 98, 113] ↑ time to first thrombus >20 µm,[54, 96, 113] ↑ time to first thrombus >30 µm,[98] ↑ % thrombi forming small channels[113] - ***Adamts13*-KO**: ↑ rate of thrombus formation,[23, 53] ↑ occurrence of occlusive thrombi,[53] ↑ plt deposition 2-3 min post-injury,[108] ↓ time to occlusion,[95, 96, 108] ↓ time to first thrombus >30 µm,[95, 108] ↓ time to first thrombus >20 µm[96] - **anti-VWFA1**: ↑ time to occlusion[33] - **anti-VWFA3**: ↑ time to occlusion, ↑ time to formation of thrombus >30 µm[76] - **rADAMTS13**: ↑ time to occlusion,[78, 108] ↑ time to formation of thrombus >30 µm[78] - **Allele-selective *Vwf* silencing**: ↑ time to occlusion, ↓ non-occlusive thrombus area[142] - **With expression of VWF mutants:**   - VWD Type 2B mutant (V1316M): ↑ time to occlusion,[48] ↓ occurrence of occlusion, ↓ formation of thrombus >50 µm[85]   - VWD Type 2B mutant (R1306Q): ↓ occurrence of occlusion, ↓ formation of thrombus >50 µm[85]   - L1696R or P1824H (A3 domain): ↓ occurrence of occlusion[66]   - D2509G (GP2B3a binding domain), K1362A (GPIbɑ binding domain), or VWF-collagen binding mutant: ↑ time to occlusion, ↑ time to first thrombus >30 µm[98] |
|  |  |  | Middle cerebral artery | 6 (4.2) | - **Monitoring**: 60 min,[20, 137] 70 min,[131] 120 min[42] - **Filter paper size:** 0.5 x 0.5 mm (“small”) or 0.5 x 1.5 mm (“large”)[42] | - VWF stain in thrombus,[42, 64] VWF colocalizes with plt in small downstream arteries,[137] VWF/plt aggregate formation correlates with ET-1 stain and neutrophil recruitment in ischemic tissue[137] - ***Vwf*-KO**: ↑ cerebral blood flow, ↓ infarct size, ↓ occlusion[64] - ***Adamts13*-KO**: ↓ time to first occlusion, ↓ blood flow (↑ by **rADAMTS13**), ↑ infarct size[42] - **rADAMTS13**: ↑ recanalization,[20] ↑ cerebral blood flow (WT,[42] GoF-ADAMTS13,[20] caADAMTS13[137]), ↓ infarct volume (WT,[20, 42] caADAMTS13[137]), ↓ infarct size,[42] ↓ neutrophil infiltration (WT,[118] caADAMTS13[137]), ↓ VWF, fibrin, plt, and ET-1 stain in ischemic tissue (caADAMTS13[137]), ↓ microglial activation (caADAMTS13[137]) - **anti-VWFA1**: ↓ occlusion, ↓ infarct size, ↓ VWF stain in thrombus, ↓ VWF-rich portion of thrombus, ↓ thrombus size, ↓ % occlusive thrombi on histology, ↑ cerebral blood flow[64] - **VWF-targeted uPA**: ↓ infarct volume[131] |
|  |  |  | Cremasteric arteriole(s) | 4 (2.8) | - **Vessel size**: 30-60 µm,[60] 7.5-18 µm,[73] 50-75 µm[84] - **Filter paper size**: 1 x 1 mm[73, 80, 84] | - ***Vwf*-KO**: ↓ extent of occlusion,[60] ↑ time to occlusion, ↓ plt accumulation[84] - **With expression of VWF mutants:**   - R1597W or VWFY1605A/M1606A: ↑ time to occlusion[73]   - Y1605A/M1606A: ↑ plt accumulation[73]   - VWD Type 1 mutant Y1605A/M1606A: ↓ plt accumulation, ↑ time to occlusion[80]   - VWD Type 2B mutants R1306W, R1341Q, and V1316M: ↑ time to occlusion, ↓ plt accumulation[84] |
|  |  | Venous | Mesenteric venule(s) | 8 (5.6) | - **Ferric chloride concentration**: 2.5%,[79] 7.5%,[62, 85] 10%[32, 33, 48, 103, 116] - **Ferric chloride application method**: filter paper,[62, 85, 103, 116] topical[33, 48, 79] - **Filter paper application time**: 1.5 min,[62] 2 min,[116] 5 min[85, 103] - **Filter paper size**: 0.5 x 4mm[85] - **Monitoring**: 23s,[79] 1 min,[62] 5 min,[116] 40 mi,[85, 103] 60 min[33, 48] - **Vessel size**: 180 ± 6 µm,[116] 200-250 µm,[79, 85] 200-300 µm[103] | - Fluorescent VWF in thrombus[79] - ***Vwf*-KO**: ↑ time to 50 µm, 100 µm, and 150 µm thrombus, ↑ clotting time, ↑ number of emboli, ↑ time to occlusion, ↓ clot rate, ↓ number of plt adhering,[103] ↓ plt string formation[116] - ***Adamts13*-KO**: ↑ string length,[79] ↑ string survival time[79, 116] (↓ by **rADAMTS13**[116]) - **rADAMTS13**: ↓ plt string length, ↓ % coverage by plt/leukocyte aggregates[62] - **anti-VWFA1**: ↑ time to occlusion[33] - **anti-ADAMTS13**: ↑ string survival time[32] - **VWD Type 2B mutant V1316M expression**: ↑ time to occlusion, ↑ VWF/plt string size, ↑ string survival time,[48] ↓ thrombus >50 µm formation, ↓ occlusion[85] |
|  |  |  | Dorsal skin venule(s) | 1 (0.7) | - | **rADAMTS13**: ↑ blood flow, ↓ thrombus size[71] |
|  |  | Unspecified | Mesenteric vessels | 2 (1.4) | - Pre-stimulation with epinephrine[36] - **Ferric chloride concentration**: 10%,[101] 20%[36] - **Filter paper size**: 2 x 1 mm[36] - **Application time**: 3 min,[101] 2 min[36] - **Monitoring**: 20 min,[36] 60 min[101] - **Vessel size**: 100-200 µm,[101] 3 mm[36] | - Fluorescent VWF in thrombus[36] - ***Vwf*-KO**: ↑ time to occlusion[101] |
|  | Laser | Arterial | Cremasteric arteriole(s) | 7 (4.9) | - Laser only;[12, 50, 59, 102, 106] Photochemical[34, 100] - **Sensitizing agent**: hematoporphyrin,[34] FITC-dextran[100] - **Light wavelength used for injury:** 440 nm,[102] 491 nm[34] - **Monitoring**: 2 min,[59] 3.5 min,[50] 350s,[106] 10 min,[34] 20 min[12] - **Vessel size**: 20-40 µm,[50] 40-65 µm,[102] 100 µm[100] | - ***Vwf*-KO**: ↑ time to occlusion,[34, 100] ↓ thrombus size,[59, 102] ↓ maximum thrombus size[106] - **anti-ADAMTS13 Ab expression**: ↑ thrombus elongation[50] - **rADAMTS13:** ↓ fibrin and platelet accumulation in HIT mice[12] - **With expression of VWF mutants:**   - VWD Type 2N mutants (R763A, R854Q, or R816W): ↓ thrombus size, ↓ thrombus volume, ↓ number of thrombi, ↑ number of small emboli[34]   - R1326H (disrupts plt blinding): ↓ thrombus size[59]   - Human plt-binding VWF: creates a thrombus composed of human plts in contrast to WT when human plts are administered, indicating binding[102] |
|  |  |  | Mesenteric arteriole(s) | 4 (2.8) | - Laser only;[38, 46, 90] Photochemical[75] - Specified superficial and deep injuries[90] - **Light wavelength used for injury**: 365-400 nm,[38, 46] 440 nm,[90] 4 nm[75] - **Monitoring**: 20s,[75] 4 min,[38] 5 min,[46] 10 min[90] - **Vessel size**: 60-100 µm,[38, 46] 65-100 µm[90] | - ↑ VWF vascular surface expression[75] - ***Vwf*-KO**: ↓ plt adherence,[38] ↓ thrombus area (deep lesion), minimal thrombus formation (superficial lesion),[90] ↑ thrombus size, growth, and % occlusion when supplemented with WT plt (in contrast to *Vwf*-KO plt)[46] |
|  |  |  | Caecal arteriole(s) | 2 (1.4) | - **Rose-bengal dosing**: 10 mg/kg bolus + 10 mg/kg/h infusion,[105] 20 mg/kg bolus + 20 mg/kg/h infusion[114] - **Rose-bengal infusion time**: 40 min,[105] 30 min[114] - **Injury time**: 20s,[105] 40s[114] - **Vessel size**: 52±23 µm,[105] 60-100 µm[114] | - **anti-ADAMTS13**: ↓ time to occlusion compared to WT in *Tsp*-KO[105] - **rmVWFA1**: ↑ time to occlusion[114] |
|  |  |  | Cortical surface arteriole(s) | 1 (0.7) | - | **caADAMTS1**3: ↓ VWF and neutrophil staining in ischemic brain[118] |
|  |  |  | Carotid artery | 1 (0.7) | - | **rADAMTS13**: ↑ time to occlusion in HIT mice[12] |
|  |  | Venous | Caecal venule(s) | 2 (1.4) | - **Rose-bengal dosing**: 10 mg/kg bolus + 10 mg/kg/h infusion,[105] 20 mg/kg bolus + 20 mg/kg/h infusion[114] - **Rose-bengal infusion time**: 40 min,[105] 30 min[114] - **Injury time**: 20s,[105] 40s[114] - **Vessel size**: 105±32 µm,[105] 60-100 µm[114] | - **anti-ADAMTS13**: ↓ time to occlusion compared to WT in *Tsp*-KO[105] - **rmVWFA1**: ↑ time to occlusion[114] |
|  |  |  | Mesenteric venule(s) | 1 (0.7) | - | ***Adamts13*-KO**: ↑ thrombus size at 300s, fluorescent VWF in thrombus[79] |
|  |  |  | Cremasteric venule(s) | 1 (0.7) | - | ***Vwf*-KO**: ↑ time to occlusion[100] |
|  |  | Capillary | Mesenteric capillaries | 1 (0.7) | - | ↑ VWF vascular surface expression[75] |
|  | Calcium ionophore | Venous | Mesenteric venule(s) | 8 (5.6) | - **Calcium ionophore volume and concentration**: 10 µL of 30 µM,[105] 10 µL of 10 µM,[16, 81] 30 µL of 10 µM,[109, 112] 10 µM[108] - **Monitoring**: 2 min,[107] 10 min,[105, 108] 20 min[16, 81, 109] - **Vessel size**: 172 µm or 195 µm depending on genotype,[105] 25-30 µm,[107, 108] 100-200 µm,[112] 100-150 µm,[16] 120-150 µm,[81] 200-300 µm[109] | - ***Vwf*-KO**: ↓ plt adherence[109, 112] - ***Adamts13*-KO**: ↑ thrombus clearance time, ↑ thrombus formation[81] - **rADAMTS13:** ↓ number of emboli >20 µm, ↓ time to thrombus resolution, ↓ plt accumulation[16] - **anti-ADAMTS13**: ↑ plt adherence,[105, 109] ↑ thrombus formation[107, 108] |
|  |  | Unspecified | Mesenteric vessels | 1 (0.7) | - | **rmVWFA1**: ↓ plt rolling[114] |
|  | Transient occlusion using filament | Arterial | Middle cerebral artery | 17 (11.9) | - **Monofilament material**: Silicon-rubber-coated,[8, 18, 52, 89, 117, 129] siliconized,[46, 49, 67, 72, 86, 137] nylon tip rounded by paraffin,[57] nylon[74] - **Monofilament size**: 6-0,[8, 18, 46, 52, 89, 129, 137] 7-0,[49, 67, 72, 93] 0.148-0.165mm diameter[57] - **Occlusion time**: 30 min,[74, 86, 137] 45 min,[18, 49, 67] 60 min,[8, 46, 52, 72, 89, 97, 117, 129] 90 min,[57] 2 h,[93] 2 h or 4 h[55] - Inserted filament first through ECA prior to ICA[117, 137] - Permanently ligated ECA following occlusion[117] - Cerebral hemorrhaging increased by prior IP injection of D-glucose[67] | - ↑ VWF stain in ischemic tissue[18, 57] - **Intraventricular rhVWF injection**: ↑ BBB permeability (↓ by coinjection with **rADAMTS13**), ↑ hemorrhage volume[67] - ***Vwf*^+/-^:** ↓ infarct V[93] - ***Vwf*-KO**: ↑ cerebral blood flow at reperfusion,[46] ↓ infarct volume,[8, 46, 52, 89, 93, 97, 129] ↓ WBC recruitment, ↓ VWF/plt aggregates,[8] ↓ neutrophil recruitment,[8, 72] ↓ IL-6 and TNFα, ↓ MPO,[72] ↓ fibrinogen deposition,[52, 89] ↓ neurological deficit,[46, 89, 97] restricts thrombi to basal ganglia (no cortical thrombi),[97] ↓ NETs,[129] **supplementing VWF** ↑ infarct volume, ↑ neurological deficit, ↑ fibrinogen deposition[89] - **anti-VWFA1**: ↓ infarct volume, ↓ neurological deficit, ↓ immune cell recruitment, ↓ VWF/plt aggregates[8] - **plt-VWF only, or plt-VWF with *Adamts13*-KO**: ↓ neurological deficit, ↓ infarct volume, ↓ fibrin(ogen) density, ↓ neutrophil recruitment, ↓ TNFα, ↓ IL-1β, ↑ cerebral blood flow at reperfusion compared to ***Vwf*-KO**[46] - ***Adamts13*-KO**: ↑ infarct size,[49, 74, 86, 117] ↑ infarct volume (↓ with ***Vwf*-KO**),[93] ↑ cerebral edema,[117] ↑ neuronal death, ↑ plasma HMGB1, ↑ MPO,[74] ↑ neutrophil recruitment, ↑ IL-6 and TNFα,[72] ↑ neurological deficit,[86, 117] ↑ VWF-positive thrombi, ↑ inflammatory cells, ↓ cerebral blood flow[86] - **rADAMTS13**: ↑ cerebral blood flow[55] (caADAMTS13[137]), ↓ plasma HMGB1,[55] ↓ infarct volume[49, 55, 93] (caADAMTS13[137]), ↓ time to completion of tape removal test,[93] ↓ vascular leakage (caADAMTS13), ↓ peri-infarct microglial activation (caADAMTS13), ↓ >30 µm^2^ platelet aggregates in striatum (caADAMTS13),[137] ↓ neurological deficit, ↓ fibrin deposition, ↓ necrosis, ↓ pERK and NR2B, ↓ apoptosis, and ↓ autophagy in ischemic tissue (compared to tPA), ↓ apoptosis in ischemic tissue,[49] ↓ tPA-induced BBB disruption, ↓ tPA-induced hemorrhage, ↓ tPA-induced neurological deficit, ↓ tPA-evoked endothelial VEGF induction[67] |
|  | Ligation | Arterial | Common carotid | 2 (1.4) | - **Suture type**: polypropylene,[30] silk[3] - Ligated vigorously[30] - Crushed with forceps over suture[3] | Interrupting VWF unfolding ↑ blood flow, ↑ reperfusion;[3] reducing plasma VWF to 50% ↓ plt adherence to carotid[30] |
|  |  |  | External carotid artery | 1 (0.7) | - | ↑ fluorescent VWF in ligated carotid, ↑ proportion of HMW multimer, ↑ plasma VWF[6] |
|  |  | Venous | Inferior vena cava | Stenosis: 3 (2.1)  Stasis: 3 (2.1) | Stenosis:   - **Suture size**: 2-0,[11] 7-0[82] - **Suture type**: Monocryl,[11] polypropylene[82]   Stasis:   - **Suture size**: 4-0,[139] 7-0[82] - **Suture type**: polypropylene,[82] silk + clamp,[139] silk[13] - Transient stasis,[139] permanent stasis[82] | Stenosis:   - ↑ plasma VWF,[11, 128] ↑ VWF:CB activity, ↓ VWFCB:VWFAg ratio,[128] fluorescent VWF in thrombus,[82] VWF concentrated in white thrombus,[11, 128] VWF forms network in red thrombus,[11] VWF stain correlates with thrombus size; VWF in thrombus correlates with P-selectin and plt, negatively correlates with RBCs, and colocalizes with fibrinogen[128] - **anti-VWF**: ↓ thrombus development, ↓ thrombus weight, ↓ thrombus development (with LPS)[11] - **VWFA1 nanobody**: ↓ thrombus development, ↓ thrombus weight, ↓ leukocyte recruitment[11] - ***Vwf*-KO and *Vwf*^+/-^**: ↓ % mice with thrombus, ↓ thrombus length, ↓ thrombus weight[82] - ***Vwf*-KO**: ↓ leukocytes and plt at site of endothelial activation (9382),   Stasis:   - ↑ plasma VWF[13, 139] - ***Vwf*-KO**: ↓ % mice with thrombus, ↓ thrombus length, ↓ thrombus weight[82] |
|  | Cautery | Arterial | Middle cerebral artery | 1 (0.7) | - | - ↑ plasma VWF, ↑ VWF:CB (in *Cd69*-KO)[18] - **anti-VWF**: ↓ infarct V (in *Cd69*-KO)[18] |
|  | Copper cuff | Arterial | Carotid artery | 1 (0.7) | - | VWF staining in 13% of thrombus sample[110] |
|  | Polyethylene cuff and ligation | Arterial | Carotid artery | 1 (0.7) | - | ↑ plasma VWF, VWF staining flanking cuff placement (not under cuff); VWF vascular surface expression throughout vessel with ligation or cuff alone[111] |
|  | Mechanical denudation and transplant | Arterial | Aorta | 1 (0.7) | - | ↑ VWF staining in aortic segments containing thrombus[41] |
|  | Thrombin injection | Arterial | Middle cerebral artery | 1 (0.7) | - | **VWF-targeted uPA (Microlyse)**: ↑ reperfusion, ↓ lesion volume[131] |
|  | Clamp (I/R) | Venous | Cremasteric venule(s) | 1 (0.7) | - | **rADAMTS13**: ↓ leukocyte adhesion, ↑ leukocyte rolling velocity, ↑ leukocyte rolling[126] |
|  |  | **Organ** |  | | | |
| **Organ-**  **specific** | Ligation | Heart | Left anterior descending artery | 8 (5.6) | - **Suture size**: 6-0,[25] 7-0,[138] 8-0[4, 22, 69, 70, 121] - **Suture type**: nylon,[4, 69, 70, 121] polypropylene,[22, 25] silk[138] - **Time with chest closed before tightening**: 5 days,[126] 5-7 days,[4, 25, 121] 7 days[70] - **Tension time**: 30 min,[69] 35 or 40 min,[22, 126] 40 min,[4, 25, 121] 60 min,[70] permanent[138] - Open-chest tension[69, 138] | - ↑ VWF and plt GPIbɑ molecular signal in ascending aorta and proximal aortic arch,[4] ↑ VWF stain in myocardium[22, 25, 138] (↓ by **rADAMTS13**[22]), ↑ VWF molecular signal in risk area,[126] ↑ VWF antigen (↑ in *Nlrp3*-KO with NLRP3+ neutrophils)[138] - ***Vwf*-KO**: ↓ infarct size, ↓ plasma troponin T, ↓ myocyte apoptosis, ↓ neutrophil recruitment[69] - ***Adamts13*-KO**: ↑ infarct size,[22, 69, 70] ↑ plasma troponin T, ↑ neutrophil recruitment, ↑ myocyte apoptosis,[69] ↑ VWF/plt stain in myocardium, ↓ stroke volume[22] - **anti-VWF:** ↓ infarct size, ↓ plasma troponin T[70] - **rADAMTS13**: ↑ recovery of LV function, ↑ risk area reflow in hyperlipidemia,[126] ↑ myocardial microvascular blood flow,[22] ↓ infarct size,[22, 121, 126] ↓ GPIbɑ stain in myocardium in atherosclerosis,[25] ↓ VWF, GPIbɑ, P-selectin and VCAM-1 molecular signal in proximal aorta, ↓ CD41 staining in aortic root,[121] ↓ VWF, GPIbɑ, and CD18 (leukocyte) molecular signal in risk area, ↓ plt, neutrophil, monocyte, and MMP-9 stain in myocardium, ↓ adverse remodeling of myocardium, ↓ plt/leukocyte complexes in hyperlipidemia[126] |
|  |  | Kidney | Renal artery (unilateral; “hanging-weight”) | 1 (0.7) | - | - ULVWF adheres to RBCs and occludes renal capillaries[26] - ***Vwf*-KO**: ↓ RBC adherence to inner stripe, ↓ acute tubular necrosis[26] |
|  | Clamp (I/R) | Liver | Hepatic artery and portal vein | 1 (0.7) | - | - ***Vwf*-KO or rADAMTS13**: ↓ ALT, ↓ hepatic neutrophil infiltration, ↑ hepatic blood flow[27] - ***Vwf*-KO**: ↓ rolling, adhering, and plugging WBCs, ↓ WBC-plt adherence[27] |
|  |  | Kidney | Renal arteries and veins (bilateral) | 1 (0.7) | - | - ↓ plasma and renal ADAMTS13, ↑ renal VWF staining, ↑ renal ULVWF dimers[40] - ***Adamts13*-KO**: ↑ renal ULVWF dimers, ↑ renal neutrophil infiltration, ↑ tubular injury (↓ with **anti-VWF**), ↑ creatinine (↓ with **anti-VWF**), ↑ renal inflammatory markers (↓ with **anti-VWF**), ↓ renal CD31 staining[40] |
|  | **Condition induced** | **Method of induction** |  | | | |
| **Systemic** | Congenital TTP | *Adamts13*-KO with VWF administration | - | 12 (8.4) | - **VWF source**: recombinant,[16, 29, 47] recombinant murine,[53] recombinant human,[2, 31, 37, 44, 58, 77] purified human[43, 125] - **VWF dose**: 250 U/kg,[29] 2000 U/kg,[16, 37, 44, 47, 58, 77] 2250 U/kg,[2, 31] 5 mg/kg,[43, 125] 5-10 µg/g[53] | - ↓ plt[2, 29, 31, 37, 43, 44, 53, 77] (↑ by **VWF-targeted uPA**[2]), ↓ Hb,[31, 37, 44] ↓ hematocrit, ↓ GPIbɑ stain in liver,[29] ↓ body weight,[37, 44] ↓ LV function, ↓ cardiac perfusion,[44, 125] ↑ LDH[2, 29, 31, 37] (↓ by **VWF-targeted uPA**[2]), ↑ PAI-1 (↓ by **VWF-targeted uPA**),[2] ↑ number of plt aggregates in pial microvasculature,[16] ↑ myocardial necrosis,[31, 44, 77] ↑ myocardial hemorrhaging,[44, 77] ↑ myocardial neutrophil infiltration,[77] ↑ VCAM-1 in heart tissue, ↑ cardiomyocyte destruction with edema and lymphocyte infiltration, ↑ schistocytosis,[44] ↑ VWF/plt molecular signal in myocardial microcirculation,[125] ↑ occlusive thrombi in brain, heart, lungs, kidneys, and liver[53] - **rADAMTS13**: ↑ plt,[47, 77] ↑ Hb, ↑ body weight,[77] ↓ LDH,[47, 77] ↓ schistocytosis,[47] ↓ myocardial necrosis, hemorrhaging, and neutrophil infiltration,[77] ↓ microvascular occlusion[16] - **Prior *Adamts13* gene therapy**: ↑ plt,[37, 58, 77] ↑ Hb, ↑ body weight, ↓ LDH[37] - **Fresh frozen plasma**: ↑ plt, ↓ LDH, ↓ schistocytosis[47] |
|  |  | *Adamts13*-KO with Shiga toxin administration | - | 5 (3.5) | - **Shiga toxin type**: Stx-2,[53, 68, 96, 109] Stx-2B[83] - **Shiga toxin dose**: 20-250 pg/g,[53] 125 pg/g,[109] 250 pg/g,[68, 96] 1.25 ng/g[83] | - ↓ plt,[53, 68, 83, 109] ↓ Hb,[83, 109] ↓ survival,[109] ↑ occlusive thrombi in brain, heart, kidneys, lungs and liver,[53, 68] ↑ schistocytosis,[83, 109] ↑ reticulocytosis,[83] ↑ VWF-rich, fibrin-poor hyaline thrombi in brain, heart, and kidneys[109] - ***Vwf*-KO or *Vwf*^+/-^**: protected from ↓ plt[96] |
|  | Acquired TTP | Neutralizing anti-ADAMTS13 Ab administration with VWF trigger | - | 4 (2.8) | - **Antibody dose**: 1.25 mg/kg,[32, 45, 136] 10 µg/g[53] - **Time between antibody and VWF administration**: 8h,[53] 24h[32, 45, 136] - **VWF source**: human purified,[136] recombinant murine,[53] recombinant human,[45] recombinant[32] - **VWF dose**: 2000 U/kg,[32] 500 U/kg,[45] 5-10 µg/g,[53] 5 mg/kg[136] | ↓ plt,[32, 45, 53, 136] ↓ Hb, ↑ PAP, ↑ PAI-1,[32] ↑ LDH,[45, 136] ↑ occlusive thrombi in brain, heart, lungs, liver, and kidneys,[53] ↑ pulmonary VWF/plt aggregates[136] |
|  |  | Hydrodynamic injection of cDNA encoding anti-ADAMTS13 Ab variable region with Shiga toxin trigger | - | 1 (0.7) | - | ↓ survival, ↑ schistocytosis, ↑ thrombi in brain, heart, and kidneys[50] |
|  | TTP | Hydrodynamic injection of cDNA encoding cleavage-resistant VWF (VWF/p.S1494C-p.A1534C) | - | 1 (0.7) | - | ↓ plt, ↑ schistocytosis, ↑ BUN, ↑ creatinine, ↑ renal VWF/plt aggregates[63] |
|  | Spontaneous TMA | Homozygous *cfh* mutation, or heterozygeous *cfh* mutation with *Adamts13*-KO | - | 1 (0.7) | - | - ↑ plasma VWF, ↓ proportion of HMW VWF[14] - ***Adamts13*-KO with *cfh* mutation**: ↓ plt, ↓ survival, ↑ schistocytosis, ↑ BUN, ↑ creatinine, ↑ LDH[14] - ***Adamts13*-KO** **alone**: ↑ microthrombi in brain, heart, kidneys, and intestines[14] |
|  | Induced TMA | Administration of adenovirus encoding VEGF inhibitor (*sFlt-1*) | - | 1 (0.7) | - | - ***Adamts13*-KO**: ↓ plt, ↓ Hb, ↓ open glomerular capillary volume, ↑ schistocytosis, ↑ reticulocytosis, ↑ plasma VWF, ↑ VWF-positive thrombi in heart, liver, and kidneys, ↑ ACR, ↑ BP (all reversed by **rADAMTS13**)[51] - **rADAMTS13**: ↓ glomerular area in *Adamts13*-KO and WT mice[51] |
|  | Venous microvascular thrombosis | Collagen-  epinephrine | - | 2 (1.4) | - **Outcome assessment**: After 5 minutes,[95] after 15 minutes[104] | ***Adamts13*-KO**: ↓ plt counts[95, 104] |
|  | Endothelial activation | Histamine administration | Mesenteric venule(s) | 3 (2.1) | - **Route of histamine administration**: IV,[107] IP[108, 112] - **Monitoring**: 4 min,[112] 5 min[107, 108] | - **anti-ADAMTS13**: ↑ string survival (more pronounced in P-selectin-KO)[107] - ***Adamts13*-KO**: strings and aggregates form (↓ by r**ADAMTS13**)[108] - ***Vwf*-KO**: ↓ number of adhering plt[112] |
|  |  | DDAVP administration | Mesenteric venule(s) | 1 (0.7) | - | - VWF/plt strings form[79] - ***Adamts13*-KO**: ↑ total number, ↑ total length, and ↑ average length of strings[79] |
|  |  | Histone administration | Cremaster venule(s) | 1 (0.7) | - | ***Vwf*-KO**: in contrast to WT, no ↑ plt adhesion[133] |
|  |  | CD40L administration | Cremasteric microcirculation | 1 (0.7) | - | - VWF/plt strings form[10] - ***Adamts13*-KO**: ↑ number of strings, ↑ ULVWF-leukocyte colocalization[10] |

Items in bold in the outcomes column indicate alterations made to the model that led to the listed outcomes relative to control (e.g., *rADAMTS13,* administration of recombinant ADAMTS13; *rmVWFA1,* administration of recombinant mouse VWFA1 fragment; *Vwf*-KO*,* performing model on *Vwf*-KO mice). Outcomes listed without an alteration pertain to the model itself. *Anti-*, antibody against indicated target; *Ab*, antibody; -*KO,* genetic knockout mice; *VWD,* von Willebrand disease; *HIT,* heparin-induced thrombocytopenia; *Plt-VWD,* platelet-type von Willebrand disease*; plt-VWF*, *Vwf*-KO mice with platelets containing VWF; *plt*, platelet; *Hb*, hemoglobin; *V,* volume; *ACR,* albumin-creatinine ratio; *RBC,* red blood cell; *BP,* blood pressure; *LV*, left ventricle of the heart; *HMW*, high molecular weight; *ULVWF*, ultra-long VWF multimers; *BUN*, blood urea nitrogen; *LDH,* lactate dehydrogenase activity; *DDAVP,* desmopressin (1-deamino-8-D-arginine vasopressin); *TTP,* thrombotic thrombocytopenic purpura; *tPA,* tissue plasminogen activator; *uPA*, urokinase-type plasminogen activator; *caADAMTS13*, constitutively active ADAMTS13; *GoF-ADAMTS13*, gain-of-function ADAMTS13; *PAP,* plasmin-α2-antiplasmin complex; *PAI-1*, plasminogen activator inhibitor-1; NETs, neutrophil extracellular traps; *rh,* recombinant human; *rm*, recombinant mouse; *IV,* intravenous; *IP,* intraperitoneal; *I/R,* ischemia-reperfusion; *HIT*, heparin-induced thrombocytopenia.

**References for Supplement**

1.	Plautz WE, Haldeman SH, Dyer MR, Sperry JL, Guyette FX, Loughran PA, et al. Reduced cleavage of von willebrand factor by ADAMTS13 is associated with microangiopathic acute kidney injury following trauma. Blood coagulation & fibrinolysis : an international journal in haemostasis and thrombosis. 2021(a5j, 9102551).

2.	de Maat S, Clark C, Barendrecht AD, Smits S, van Kleef ND, El Otmani H, et al. Microlyse; a thrombolytic agent that targets VWF for clearance of microvascular thrombosis. Blood. 2021(a8g, 7603509).

3.	Griffin MT, Ashworth K, Hill N, von Behren J, Di Paola J, Ku DN. Negatively charged nanoparticles of multiple materials inhibit shear-induced platelet accumulation. Nanomedicine : nanotechnology, biology, and medicine. 2021;35(101233142):102405.

4.	Shentu W, Ozawa K, Nguyen TA, Wu MD, Packwood W, Xie A, et al. Echocardiographic Molecular Imaging of the Effect of Anticytokine Therapy for Atherosclerosis. Journal of the American Society of Echocardiography : official publication of the American Society of Echocardiography. 2021;34(4):433–42.e3.

5.	Cui W, Wu X, Feng D, Luo J, Shi Y, Guo W, et al. Acrolein Induces Systemic Coagulopathy via Autophagy-dependent Secretion of von Willebrand Factor in Mice after Traumatic Brain Injury. Neuroscience bulletin. 2021;37(8):1160–75.

6.	Zhu J-J, Jiang Z-T, Liu C, Xi Y-F, Wang J, Yang F-F, et al. VAMP3 and SNAP23 as Potential Targets for Preventing the Disturbed Flow-Accelerated Thrombus Formation. Frontiers in cell and developmental biology. 2020;8(101630250):576826.

7.	Ozawa K, Muller MA, Varlamov O, Tavori H, Packwood W, Mueller PA, et al. Proteolysis of Von Willebrand Factor Influences Inflammatory Endothelial Activation and Vascular Compliance in Atherosclerosis. JACC Basic to translational science. 2020;5(10):1017–28.

8.	Denorme F, Martinod K, Vandenbulcke A, Denis CV, Lenting PJ, Deckmyn H, et al. The von Willebrand Factor A1 domain mediates thromboinflammation, aggravating ischemic stroke outcome in mice. Haematologica. 2021;106(3):819–28.

9.	Jin X, Jin C, Nakamura K, Jin T, Xin M, Wan Y, et al. Increased dipeptidyl peptidase-4 accelerates chronic stress-related thrombosis in a mouse carotid artery model. Journal of hypertension. 2020;38(8):1504–13.

10.	Tahir S, Wagner AH, Dietzel S, Mannell H, Pircher J, Weckbach LT, et al. Endothelial CD40 Mediates Microvascular von Willebrand Factor-Dependent Platelet Adhesion Inducing Inflammatory Venothrombosis in ADAMTS13 Knockout Mice. Thrombosis and haemostasis. 2020;120(3):466–76.

11.	Michels A, Dwyer CN, Mewburn J, Nesbitt K, Kawecki C, Lenting P, et al. von Willebrand Factor Is a Critical Mediator of Deep Vein Thrombosis in a Mouse Model of Diet-Induced Obesity. Arteriosclerosis, thrombosis, and vascular biology. 2020;40(12):2860–74.

12.	Johnston I, Sarkar A, Hayes V, Koma GT, Arepally GM, Chen J, et al. Recognition of PF4-VWF complexes by heparin-induced thrombocytopenia antibodies contributes to thrombus propagation. Blood. 2020;135(15):1270–80.

13.	Li D, Zhang X, Zhang H, Li X. Synergic effect of GPIBA and von Willebrand factor in pathogenesis of deep vein thrombosis. Vascular. 2020;28(3):309–13.

14.	Zheng L, Zhang D, Cao W, Song W-C, Zheng XL. Synergistic effects of ADAMTS13 deficiency and complement activation in pathogenesis of thrombotic microangiopathy. Blood. 2019;134(13):1095–105.

15.	Latifi Y, Moccetti F, Wu M, Xie A, Packwood W, Qi Y, et al. Thrombotic microangiopathy as a cause of cardiovascular toxicity from the BCR-ABL1 tyrosine kinase inhibitor ponatinib. Blood. 2019;133(14):1597–606.

16.	Adili R, Holinstat M. Formation and Resolution of Pial Microvascular Thrombosis in a Mouse Model of Thrombotic Thrombocytopenic Purpura. Arteriosclerosis, thrombosis, and vascular biology. 2019;39(9):1817–30.

17.	Nimjee SM, Dornbos D, 3rd, Pitoc GA, Wheeler DG, Layzer JM, Venetos N, et al. Preclinical Development of a vWF Aptamer to Limit Thrombosis and Engender Arterial Recanalization of Occluded Vessels. Molecular therapy : the journal of the American Society of Gene Therapy. 2019;27(7):1228–41.

18.	Brait VH, Miro-Mur F, Perez-de-Puig I, Notario L, Hurtado B, Pedragosa J, et al. CD69 Plays a Beneficial Role in Ischemic Stroke by Dampening Endothelial Activation. Circulation research. 2019;124(2):279–91.

19.	Slobodianuk TL, Kochelek C, Foeckler J, Kalloway S, Weiler H, Flood VH. Defective collagen binding and increased bleeding in a murine model of von Willebrand disease affecting collagen IV binding. Journal of thrombosis and haemostasis : JTH. 2019;17(1):63–71.

20.	South K, Denorme F, Salles C, II, De Meyer SF, Lane DA. Enhanced activity of an ADAMTS-13 variant (R568K/F592Y/R660K/Y661F/Y665F) against platelet agglutination in vitro and in a murine model of acute ischemic stroke. J Thromb Haemost. 2018;16(11):2289–99.

21.	Wan H, Wang Y, Ai J, Brathwaite S, Ni H, Macdonald RL, et al. Role of von Willebrand factor and ADAMTS-13 in early brain injury after experimental subarachnoid hemorrhage. Journal of thrombosis and haemostasis : JTH. 2018;16(7):1413–22.

22.	Ozawa K, Packwood W, Varlamov O, Qi Y, Xie A, Wu MD, et al. Molecular Imaging of VWF (von Willebrand Factor) and Platelet Adhesion in Postischemic Impaired Microvascular Reflow. Circulation Cardiovascular imaging. 2018;11(11):e007913.

23.	Abdelgawwad MS, Cao W, Zheng L, Kocher NK, Williams LA, Zheng XL. Transfusion of Platelets Loaded With Recombinant ADAMTS13 (A Disintegrin and Metalloprotease With Thrombospondin Type 1 Repeats-13) Is Efficacious for Inhibiting Arterial Thrombosis Associated With Thrombotic Thrombocytopenic Purpura. Arteriosclerosis, thrombosis, and vascular biology. 2018;38(11):2731–43.

24.	Kanaji S, Orje JN, Kanaji T, Kamikubo Y, Morodomi Y, Chen Y, et al. Humanized GPIbalpha-von Willebrand factor interaction in the mouse. Blood advances. 2018;2(19):2522–32.

25.	Moccetti F, Brown E, Xie A, Packwood W, Qi Y, Ruggeri Z, et al. Myocardial Infarction Produces Sustained Proinflammatory Endothelial Activation in Remote Arteries. Journal of the American College of Cardiology. 2018;72(9):1015–26.

26.	Nicolay JP, Thorn V, Daniel C, Amann K, Siraskar B, Lang F, et al. Cellular stress induces erythrocyte assembly on intravascular von Willebrand factor strings and promotes microangiopathy. Scientific reports. 2018;8(1):10945.

27.	Urisono Y, Sakata A, Matsui H, Kasuda S, Ono S, Yoshimoto K, et al. Von Willebrand Factor Aggravates Hepatic Ischemia-Reperfusion Injury by Promoting Neutrophil Recruitment in Mice. Thromb Haemost. 2018;118(4):700–8.

28.	Doddapattar P, Dhanesha N, Chorawala MR, Tinsman C, Jain M, Nayak MK, et al. Endothelial Cell-Derived Von Willebrand Factor, But Not Platelet-Derived, Promotes Atherosclerosis in Apolipoprotein E-Deficient Mice. Arteriosclerosis, thrombosis, and vascular biology. 2018;38(3):520–8.

29.	Geys L, Roose E, Scroyen I, Rottensteiner H, Tersteeg C, Hoylaerts MF, et al. Platelet rescue by macrophage depletion in obese ADAMTS-13-deficient mice at risk of thrombotic thrombocytopenic purpura. Journal of thrombosis and haemostasis : JTH. 2018;16(1):150–63.

30.	Jackel S, Kiouptsi K, Lillich M, Hendrikx T, Khandagale A, Kollar B, et al. Gut microbiota regulate hepatic von Willebrand factor synthesis and arterial thrombus formation via Toll-like receptor-2. Blood. 2017;130(4):542–53.

31.	Tersteeg C, Roodt J, Van Rensburg WJ, Dekimpe C, Vandeputte N, Pareyn I, et al. N-acetylcysteine in preclinical mouse and baboon models of thrombotic thrombocytopenic purpura. Blood. 2017;129(8):1030–8.

32.	Tersteeg C, Joly BS, Gils A, Lijnen R, Deckmyn H, Declerck PJ, et al. Amplified endogenous plasmin activity resolves acute thrombotic thrombocytopenic purpura in mice. Journal of thrombosis and haemostasis : JTH. 2017;15(12):2432–42.

33.	Ayme G, Adam F, Legendre P, Bazaa A, Proulle V, Denis CV, et al. A Novel Single-Domain Antibody Against von Willebrand Factor A1 Domain Resolves Leukocyte Recruitment and Vascular Leakage During Inflammation-Brief Report. Arteriosclerosis, thrombosis, and vascular biology. 2017;37(9):1736–40.

34.	Swystun LL, Georgescu I, Mewburn J, Deforest M, Nesbitt K, Hebert K, et al. Abnormal von Willebrand factor secretion, factor VIII stabilization and thrombus dynamics in type 2N von Willebrand disease mice. Journal of thrombosis and haemostasis : JTH. 2017;15(8):1607–19.

35.	Dhanesha N, Doddapattar P, Chorawala MR, Nayak MK, Kokame K, Staber JM, et al. ADAMTS13 Retards Progression of Diabetic Nephropathy by Inhibiting Intrarenal Thrombosis in Mice. Arteriosclerosis, thrombosis, and vascular biology. 2017;37(7):1332–8.

36.	Han X, Li P, Yang Z, Huang X, Wei G, Sun Y, et al. Zyxin regulates endothelial von Willebrand factor secretion by reorganizing actin filaments around exocytic granules. Nature communications. 2017;8(101528555):14639.

37.	Verhenne S, Vandeputte N, Pareyn I, Izsvak Z, Rottensteiner H, Deckmyn H, et al. Long-Term Prevention of Congenital Thrombotic Thrombocytopenic Purpura in ADAMTS13 Knockout Mice by Sleeping Beauty Transposon-Mediated Gene Therapy. Arteriosclerosis, thrombosis, and vascular biology. 2017;37(5):836–44.

38.	Prakash P, Nayak MK, Chauhan AK. P-selectin can promote thrombus propagation independently of both von Willebrand factor and thrombospondin-1 in mice. Journal of thrombosis and haemostasis : JTH. 2017;15(2):388–94.

39.	Geys L, Bauters D, Roose E, Tersteeg C, Vanhoorelbeke K, Hoylaerts MF, et al. ADAMTS13 deficiency promotes microthrombosis in a murine model of diet-induced liver steatosis. Thrombosis and haemostasis. 2017;117(1):19–26.

40.	Kim M-G, Lim SY, Ko YS, Lee HY, Jo S-K, Cho WY. ADAMTS13-von Willebrand factor axis is involved in the pathophysiology of kidney ischaemia-reperfusion injury. Nephrology (Carlton, Vic). 2017;22(11):913–20.

41.	Kristofik N, Calabro NE, Tian W, Meng A, MacLauchlan S, Wang Y, et al. Impaired von Willebrand factor adhesion and platelet response in thrombospondin-2 knockout mice. Blood. 2016;128(12):1642–50.

42.	Denorme F, Langhauser F, Desender L, Vandenbulcke A, Rottensteiner H, Plaimauer B, et al. ADAMTS13-mediated thrombolysis of t-PA-resistant occlusions in ischemic stroke in mice. Blood. 2016;127(19):2337–45.

43.	Chung DW, Chen J, Ling M, Fu X, Blevins T, Parsons S, et al. High-density lipoprotein modulates thrombosis by preventing von Willebrand factor self-association and subsequent platelet adhesion. Blood. 2016;127(5):637–45.

44.	Le Besnerais M, Favre J, Denis CV, Mulder P, Martinet J, Nicol L, et al. Assessment of endothelial damage and cardiac injury in a mouse model mimicking thrombotic thrombocytopenic purpura. Journal of thrombosis and haemostasis : JTH. 2016;14(10):1917–30.

45.	Deforche L, Tersteeg C, Roose E, Vandenbulcke A, Vandeputte N, Pareyn I, et al. Generation of Anti-Murine ADAMTS13 Antibodies and Their Application in a Mouse Model for Acquired Thrombotic Thrombocytopenic Purpura. PloS one. 2016;11(8):e0160388.

46.	Dhanesha N, Prakash P, Doddapattar P, Khanna I, Pollpeter MJ, Nayak MK, et al. Endothelial Cell-Derived von Willebrand Factor Is the Major Determinant That Mediates von Willebrand Factor-Dependent Acute Ischemic Stroke by Promoting Postischemic Thrombo-Inflammation. Arteriosclerosis, thrombosis, and vascular biology. 2016;36(9):1829–37.

47.	Kopic A, Benamara K, Piskernik C, Plaimauer B, Horling F, Hobarth G, et al. Preclinical assessment of a new recombinant ADAMTS-13 drug product (BAX930) for the treatment of thrombotic thrombocytopenic purpura. J Thromb Haemost. 2016;14(7):1410–9.

48.	Adam F, Casari C, Prevost N, Kauskot A, Loubiere C, Legendre P, et al. A genetically-engineered von Willebrand disease type 2B mouse model displays defects in hemostasis and inflammation. Scientific reports. 2016;6(101563288):26306.

49.	Fan M, Xu H, Wang L, Luo H, Zhu X, Cai P, et al. Tissue Plasminogen Activator Neurotoxicity is Neutralized by Recombinant ADAMTS 13. Sci Rep. 2016;6:25971.

50.	Ostertag EM, Bdeir K, Kacir S, Thiboutot M, Gulendran G, Yunk L, et al. ADAMTS13 autoantibodies cloned from patients with acquired thrombotic thrombocytopenic purpura: 2. Pathogenicity in an animal model. Transfusion. 2016;56(7):1775–85.

51.	Erpenbeck L, Demers M, Zsengeller ZK, Gallant M, Cifuni SM, Stillman IE, et al. ADAMTS13 Endopeptidase Protects against Vascular Endothelial Growth Factor Inhibitor-Induced Thrombotic Microangiopathy. J Am Soc Nephrol. 2016;27(1):120–31.

52.	Verhenne S, Denorme F, Libbrecht S, Vandenbulcke A, Pareyn I, Deckmyn H, et al. Platelet-derived VWF is not essential for normal thrombosis and hemostasis but fosters ischemic stroke injury in mice. Blood. 2015;126(14):1715–22.

53.	Pickens B, Mao Y, Li D, Siegel DL, Poncz M, Cines DB, et al. Platelet-delivered ADAMTS13 inhibits arterial thrombosis and prevents thrombotic thrombocytopenic purpura in murine models. Blood. 2015;125(21):3326–34.

54.	Prakash P, Kulkarni PP, Chauhan AK. Thrombospondin 1 requires von Willebrand factor to modulate arterial thrombosis in mice. Blood. 2015;125(2):399–406.

55.	Nakano T, Irie K, Hayakawa K, Sano K, Nakamura Y, Tanaka M, et al. Delayed treatment with ADAMTS13 ameliorates cerebral ischemic injury without hemorrhagic complication. Brain Res. 2015;1624:330–5.

56.	Shim CY, Liu YN, Atkinson T, Xie A, Foster T, Davidson BP, et al. Molecular Imaging of Platelet-Endothelial Interactions and Endothelial von Willebrand Factor in Early and Mid-Stage Atherosclerosis. Circulation Cardiovascular imaging. 2015;8(7):e002765.

57.	Li T-T, Fan M-L, Hou S-X, Li X-Y, Barry DM, Jin H, et al. A novel snake venom-derived GPIb antagonist, anfibatide, protects mice from acute experimental ischaemic stroke and reperfusion injury. British journal of pharmacology. 2015;172(15):3904–16.

58.	De Cock E, Hermans C, De Raeymaecker J, De Ceunynck K, De Maeyer B, Vandeputte N, et al. The novel ADAMTS13-p.D187H mutation impairs ADAMTS13 activity and secretion and contributes to thrombotic thrombocytopenic purpura in mice. Journal of thrombosis and haemostasis : JTH. 2015;13(2):283–92.

59.	Chen J, Zhou H, Diacovo A, Zheng XL, Emsley J, Diacovo TG. Exploiting the kinetic interplay between GPIbalpha-VWF binding interfaces to regulate hemostasis and thrombosis. Blood. 2014;124(25):3799–807.

60.	Shida Y, Rydz N, Stegner D, Brown C, Mewburn J, Sponagle K, et al. Analysis of the role of von Willebrand factor, platelet glycoprotein VI-, and alpha2beta1-mediated collagen binding in thrombus formation. Blood. 2014;124(11):1799–807.

61.	Xu X, Wang C, Wu Y, Houck K, Hilton T, Zhou A, et al. Conformation-dependent blockage of activated VWF improves outcomes of traumatic brain injury in mice. Blood. 2021;137(4):544–55.

62.	Bao J, Xiao J, Mao Y, Zheng XL. Carboxyl terminus of ADAMTS13 directly inhibits platelet aggregation and ultra large von Willebrand factor string formation under flow in a free-thiol-dependent manner. Arteriosclerosis, thrombosis, and vascular biology. 2014;34(2):397–407.

63.	Morioka Y, Casari C, Wohner N, Cho S, Kurata S, Kitano A, et al. Expression of a structurally constrained von Willebrand factor variant triggers acute thrombotic thrombocytopenic purpura in mice. Blood. 2014;123(21):3344–53.

64.	Le Behot A, Gauberti M, Martinez De Lizarrondo S, Montagne A, Lemarchand E, Repesse Y, et al. GpIbalpha-VWF blockade restores vessel patency by dissolving platelet aggregates formed under very high shear rate in mice. Blood. 2014;123(21):3354–63.

65.	Vergouwen MDI, Knaup VL, Roelofs JJTH, de Boer OJ, Meijers JCM. Effect of recombinant ADAMTS-13 on microthrombosis and brain injury after experimental subarachnoid hemorrhage. Journal of thrombosis and haemostasis : JTH. 2014;12(6):943–7.

66.	Legendre P, Navarrete A-M, Rayes J, Casari C, Boisseau P, Ternisien C, et al. Mutations in the A3 domain of von Willebrand factor inducing combined qualitative and quantitative defects in the protein. Blood. 2013;121(11):2135–43.

67.	Wang L, Fan W, Cai P, Fan M, Zhu X, Dai Y, et al. Recombinant ADAMTS13 reduces tissue plasminogen activator-induced hemorrhage after stroke in mice. Ann Neurol. 2013;73(2):189–98.

68.	Zheng L, Mao Y, Abdelgawwad MS, Kocher NK, Li M, Dai X, et al. Therapeutic efficacy of the platelet glycoprotein Ib antagonist anfibatide in murine models of thrombotic thrombocytopenic purpura. Blood advances. 2016;1(101698425):75–83.

69.	Gandhi C, Motto DG, Jensen M, Lentz SR, Chauhan AK. ADAMTS13 deficiency exacerbates VWF-dependent acute myocardial ischemia/reperfusion injury in mice. Blood. 2012;120(26):5224–30.

70.	De Meyer SF, Savchenko AS, Haas MS, Schatzberg D, Carroll MC, Schiviz A, et al. Protective anti-inflammatory effect of ADAMTS13 on myocardial ischemia/reperfusion injury in mice. Blood. 2012;120(26):5217–23.

71.	Crescente M, Thomas GM, Demers M, Voorhees JR, Wong SL, Ho-Tin-Noe B, et al. ADAMTS13 exerts a thrombolytic effect in microcirculation. Thrombosis and haemostasis. 2012;108(3):527–32.

72.	Khan MM, Motto DG, Lentz SR, Chauhan AK. ADAMTS13 reduces VWF-mediated acute inflammation following focal cerebral ischemia in mice. Journal of thrombosis and haemostasis : JTH. 2012;10(8):1665–71.

73.	Pruss CM, Golder M, Bryant A, Hegadorn C, Haberichter S, Lillicrap D. Use of a mouse model to elucidate the phenotypic effects of the von Willebrand factor cleavage mutants, Y1605A/M1606A and R1597W. Journal of thrombosis and haemostasis : JTH. 2012;10(5):940–50.

74.	Fujioka M, Nakano T, Hayakawa K, Irie K, Akitake Y, Sakamoto Y, et al. ADAMTS13 gene deletion enhances plasma high-mobility group box1 elevation and neuroinflammation in brain ischemia-reperfusion injury. Neurological sciences : official journal of the Italian Neurological Society and of the Italian Society of Clinical Neurophysiology. 2012;33(5):1107–15.

75.	Nishimura S, Manabe I, Nagasaki M, Kakuta S, Iwakura Y, Takayama N, et al. In vivo imaging visualizes discoid platelet aggregations without endothelium disruption and implicates contribution of inflammatory cytokine and integrin signaling. Blood. 2012;119(8):e45–56.

76.	Navarrete A-M, Casari C, Legendre P, Marx I, Hu J-R, Lenting PJ, et al. A murine model to characterize the antithrombotic effect of molecules targeting human von Willebrand factor. Blood. 2012;120(13):2723–32.

77.	Schiviz A, Wuersch K, Piskernik C, Dietrich B, Hoellriegl W, Rottensteiner H, et al. A new mouse model mimicking thrombotic thrombocytopenic purpura: correction of symptoms by recombinant human ADAMTS13. Blood. 2012;119(25):6128–35.

78.	Xiao J, Jin S-Y, Xue J, Sorvillo N, Voorberg J, Zheng XL. Essential domains of a disintegrin and metalloprotease with thrombospondin type 1 repeats-13 metalloprotease required for modulation of arterial thrombosis. Arteriosclerosis, thrombosis, and vascular biology. 2011;31(10):2261–9.

79.	Rybaltowski M, Suzuki Y, Mogami H, Chlebinska I, Brzoska T, Tanaka A, et al. In vivo imaging analysis of the interaction between unusually large von Willebrand factor multimers and platelets on the surface of vascular wall. Pflugers Archiv : European journal of physiology. 2011;461(6):623–33.

80.	Pruss CM, Golder M, Bryant A, Hegadorn CA, Burnett E, Laverty K, et al. Pathologic mechanisms of type 1 VWD mutations R1205H and Y1584C through in vitro and in vivo mouse models. Blood. 2011;117(16):4358–66.

81.	Chen J, Reheman A, Gushiken FC, Nolasco L, Fu X, Moake JL, et al. N-acetylcysteine reduces the size and activity of von Willebrand factor in human plasma and mice. The Journal of clinical investigation. 2011;121(2):593–603.

82.	Brill A, Fuchs TA, Chauhan AK, Yang JJ, De Meyer SF, Kollnberger M, et al. von Willebrand factor-mediated platelet adhesion is critical for deep vein thrombosis in mouse models. Blood. 2011;117(4):1400–7.

83.	Huang J, Motto DG, Bundle DR, Sadler JE. Shiga toxin B subunits induce VWF secretion by human endothelial cells and thrombotic microangiopathy in ADAMTS13-deficient mice. Blood. 2010;116(18):3653–9.

84.	Golder M, Pruss CM, Hegadorn C, Mewburn J, Laverty K, Sponagle K, et al. Mutation-specific hemostatic variability in mice expressing common type 2B von Willebrand disease substitutions. Blood. 2010;115(23):4862–9.

85.	Rayes J, Hollestelle MJ, Legendre P, Marx I, de Groot PG, Christophe OD, et al. Mutation and ADAMTS13-dependent modulation of disease severity in a mouse model for von Willebrand disease type 2B. Blood. 2010;115(23):4870–7.

86.	Fujioka M, Hayakawa K, Mishima K, Kunizawa A, Irie K, Higuchi S, et al. ADAMTS13 gene deletion aggravates ischemic brain damage: a possible neuroprotective role of ADAMTS13 by ameliorating postischemic hypoperfusion. Blood. 2010;115(8):1650–3.

87.	Hemmeryckx B, Van Hove CE, Fransen P, Emmerechts J, Kauskot A, Bult H, et al. Progression of the prothrombotic state in aging Bmal1-deficient mice. Arteriosclerosis, thrombosis, and vascular biology. 2011;31(11):2552–9.

88.	Eckly A, Hechler B, Freund M, Zerr M, Cazenave JP, Lanza F, et al. Mechanisms underlying FeCl3-induced arterial thrombosis. Journal of thrombosis and haemostasis : JTH. 2011;9(4):779–89.

89.	De Meyer SF, Schwarz T, Deckmyn H, Denis CV, Nieswandt B, Stoll G, et al. Binding of von Willebrand factor to collagen and glycoprotein Ibalpha, but not to glycoprotein IIb/IIIa, contributes to ischemic stroke in mice--brief report. Arteriosclerosis, thrombosis, and vascular biology. 2010;30(10):1949–51.

90.	Hechler B, Nonne C, Eckly A, Magnenat S, Rinckel JY, Denis CV, et al. Arterial thrombosis: relevance of a model with two levels of severity assessed by histologic, ultrastructural and functional characterization. Journal of thrombosis and haemostasis : JTH. 2010;8(1):173–84.

91.	Dieude M, Gillis MA, Theoret JF, Thorin E, Lajoie G, Levine JS, et al. Autoantibodies to heat shock protein 60 promote thrombus formation in a murine model of arterial thrombosis. Journal of thrombosis and haemostasis : JTH. 2009;7(4):710–9.

92.	Guerrero JA, Kyei M, Russell S, Liu J, Gartner TK, Storrie B, et al. Visualizing the von Willebrand factor/glycoprotein Ib-IX axis with a platelet-type von Willebrand disease mutation. Blood. 2009;114(27):5541–6.

93.	Zhao BQ, Chauhan AK, Canault M, Patten IS, Yang JJ, Dockal M, et al. von Willebrand factor-cleaving protease ADAMTS13 reduces ischemic brain injury in experimental stroke. Blood. 2009;114(15):3329–34.

94.	Laje P, Shang D, Cao W, Niiya M, Endo M, Radu A, et al. Correction of murine ADAMTS13 deficiency by hematopoietic progenitor cell-mediated gene therapy. Blood. 2009;113(10):2172–80.

95.	Banno F, Chauhan AK, Kokame K, Yang J, Miyata S, Wagner DD, et al. The distal carboxyl-terminal domains of ADAMTS13 are required for regulation of in vivo thrombus formation. Blood. 2009;113(21):5323–9.

96.	Chauhan AK, Walsh MT, Zhu G, Ginsburg D, Wagner DD, Motto DG. The combined roles of ADAMTS13 and VWF in murine models of TTP, endotoxemia, and thrombosis. Blood. 2008;111(7):3452–7.

97.	Kleinschnitz C, De Meyer SF, Schwarz T, Austinat M, Vanhoorelbeke K, Nieswandt B, et al. Deficiency of von Willebrand factor protects mice from ischemic stroke. Blood. 2009;113(15):3600–3.

98.	Marx I, Christophe OD, Lenting PJ, Rupin A, Vallez M-O, Verbeuren TJ, et al. Altered thrombus formation in von Willebrand factor-deficient mice expressing von Willebrand factor variants with defective binding to collagen or GPIIbIIIa. Blood. 2008;112(3):603–9.

99.	Niiya M, Endo M, Shang D, Zoltick PW, Muvarak NE, Cao W, et al. Correction of ADAMTS13 deficiency by in utero gene transfer of lentiviral vector encoding ADAMTS13 genes. Molecular therapy : the journal of the American Society of Gene Therapy. 2009;17(1):34–41.

100.	Patel KN, Soubra SH, Bellera RV, Dong J-F, McMullen CA, Burns AR, et al. Differential role of von Willebrand factor and P-selectin on microvascular thrombosis in endotoxemia. Arteriosclerosis, thrombosis, and vascular biology. 2008;28(12):2225–30.

101.	De Meyer SF, Vandeputte N, Pareyn I, Petrus I, Lenting PJ, Chuah MKL, et al. Restoration of plasma von Willebrand factor deficiency is sufficient to correct thrombus formation after gene therapy for severe von Willebrand disease. Arteriosclerosis, thrombosis, and vascular biology. 2008;28(9):1621–6.

102.	Chen J, Tan K, Zhou H, Lo H-F, Tronik-Le Roux D, Liddington RC, et al. Modifying murine von Willebrand factor A1 domain for in vivo assessment of human platelet therapies. Nature biotechnology. 2008;26(1):114–9.

103.	Chauhan AK, Kisucka J, Lamb CB, Bergmeier W, Wagner DD. von Willebrand factor and factor VIII are independently required to form stable occlusive thrombi in injured veins. Blood. 2007;109(6):2424–9.

104.	Banno F, Kokame K, Okuda T, Honda S, Miyata S, Kato H, et al. Complete deficiency in ADAMTS13 is prothrombotic, but it alone is not sufficient to cause thrombotic thrombocytopenic purpura. Blood. 2006;107(8):3161–6.

105.	Bonnefoy A, Daenens K, Feys HB, De Vos R, Vandervoort P, Vermylen J, et al. Thrombospondin-1 controls vascular platelet recruitment and thrombus adherence in mice by protecting (sub)endothelial VWF from cleavage by ADAMTS13. Blood. 2006;107(3):955–64.

106.	Dubois C, Panicot-Dubois L, Gainor JF, Furie BC, Furie B. Thrombin-initiated platelet activation in vivo is vWF independent during thrombus formation in a laser injury model. The Journal of clinical investigation. 2007;117(4):953–60.

107.	Chauhan AK, Goerge T, Schneider SW, Wagner DD. Formation of platelet strings and microthrombi in the presence of ADAMTS-13 inhibitor does not require P-selectin or beta3 integrin. Journal of thrombosis and haemostasis : JTH. 2007;5(3):583–9.

108.	Chauhan AK, Motto DG, Lamb CB, Bergmeier W, Dockal M, Plaimauer B, et al. Systemic antithrombotic effects of ADAMTS13. The Journal of experimental medicine. 2006;203(3):767–76.

109.	Motto DG, Chauhan AK, Zhu G, Homeister J, Lamb CB, Desch KC, et al. Shigatoxin triggers thrombotic thrombocytopenic purpura in genetically susceptible ADAMTS13-deficient mice. The Journal of clinical investigation. 2005;115(10):2752–61.

110.	Sha J, McCullough B, Hart E, Nassir F, Davidson NO, Hoover-Plow J. Apo(a) promotes thrombosis in a vascular injury model by a mechanism independent of plasminogen. Journal of thrombosis and haemostasis : JTH. 2005;3(10):2281–9.

111.	Sasaki T, Kuzuya M, Cheng X-W, Nakamura K, Tamaya-Mori N, Maeda K, et al. A novel model of occlusive thrombus formation in mice. Laboratory investigation; a journal of technical methods and pathology. 2004;84(11):1526–32.

112.	Andre P, Denis CV, Ware J, Saffaripour S, Hynes RO, Ruggeri ZM, et al. Platelets adhere to and translocate on von Willebrand factor presented by endothelium in stimulated veins. Blood. 2000;96(10):3322–8.

113.	Ni H, Denis CV, Subbarao S, Degen JL, Sato TN, Hynes RO, et al. Persistence of platelet thrombus formation in arterioles of mice lacking both von Willebrand factor and fibrinogen. The Journal of clinical investigation. 2000;106(3):385–92.

114.	Oury C, Daenens K, Hu H, Toth-Zsamboki E, Bryckaert M, Hoylaerts MF. ERK2 activation in arteriolar and venular murine thrombosis: platelet receptor GPIb vs. P2X(1). JOURNAL OF THROMBOSIS AND HAEMOSTASIS. 2006;4(2):443–52.

115.	Receveur N, Maurer E, Lanza F, Gachet C, Mangin PH, Nechipurenko D, et al. Shear rate gradients promote a bi-phasic thrombus formation on weak adhesive proteins, such as fibrinogen in a von Willebrand factor-dependent manner. Haematologica. 2020;105(10):2471–83.

116.	De Maeyer B, De Meyer SF, Feys HB, Pareyn I, Vandeputte N, Deckmyn H, et al. The distal carboxyterminal domains of murine ADAMTS13 inuence proteolysis of platelet-decorated VWF strings in vivo. Journal of Thrombosis and Haemostasis. 2010;8(10):2305–12.

117.	Jiang H, Hu J, He P, Wu Y, Li F, Chen Q. ADAMTS13 deficiency exacerbates neuroinflammation by targeting matrix metalloproteinase-9 in ischemic brain injury. NeuroReport. 2024;35(7):447–56.

118.	South K, Roberts L, Gray A, Luka N, Strangward P, Coutts G, et al. Inhibition of neutrophil rolling and migration by caADAMTS13 in vitro and in mouse models of thrombosis and inflammation. Biomedicine and Pharmacotherapy. 2024;178((South, Roberts, Gray, Luka, Strangward, Coutts, Schiessl, Allan) Division of Neuroscience, School of Biological Sciences, Faculty of Biology, Medicine and Health, The University of Manchester, Manchester M13 9PT, United Kingdom(South, Roberts, Gray, Luka):117166.

119.	Bourne JH, Perrella G, El-Awaisi J, Terry LV, Tinkova V, Hogg RL, et al. Hydroxychloroquine inhibits hemolysis-induced arterial thrombosis ex vivo and improves lung perfusion in hemin-treated mice. Journal of Thrombosis and Haemostasis. 2024;22(7):2018–26.

120.	Schellenberg C, Lagrange J, Ahmed MU, Arnone D, Campoli P, Louis H, et al. The Role of Platelets and von Willebrand Factor in the Procoagulant Phenotype of Inflammatory Bowel Disease. Journal of Crohn's and Colitis. 2024;18(5):751–61.

121.	Ozawa K, Packwood W, Muller MA, Qi Y, Xie A, Varlamov O, et al. Removal of endothelial surface-associated von villebrand factor suppresses accelerate datherosclerosis after myocardial infarction. Journal of Translational Medicine. 2024;22(1):412.

122.	Shi H, Gao L, Kirby N, Shao B, Shan X, Kudo M, et al. Clearance of VWF by hepatic macrophages is critical for the protective effect of ADAMTS13 in sickle cell anemia mice. Blood. 2024;143(13):1293–309.

123.	Alavi P, Yousef Abdualla R, Brown D, Mojiri A, Nagendran J, Lewis J, et al. Aging Is Associated With Organ-Specific Alterations in the Level and Expression Pattern of von Willebrand Factor. Arteriosclerosis, Thrombosis, and Vascular Biology. 2023;43(11):2183–96.

124.	Gao D, Zhou Z, Ma R, Wu H, Nguyen T, Liu L, et al. Recombinant ADAMTS-13 Improves Survival of Mice Subjected to Endotoxemia. International Journal of Molecular Sciences. 2023;24(14):11782.

125.	Chung DW, Platten K, Ozawa K, Adili R, Pamir N, Nussdorfer F, et al. Low-density lipoprotein promotes microvascular thrombosis by enhancing von Willebrand factor self-association. Blood. 2023;142(13):1156–66.

126.	Ozawa K, Packwood W, Varlamov O, Muller M, Xie A, Wu MD, et al. Elevated LDL Cholesterol Increases Microvascular Endothelial VWF and Thromboinflammation after Myocardial Infarction. Arteriosclerosis, Thrombosis, and Vascular Biology. 2023;43(6):1041–53.

127.	An R, Man Y, Cheng K, Zhang T, Chen C, Wang F, et al. Sickle red blood cell-derived extracellular vesicles activate endothelial cells and enhance sickle red cell adhesion mediated by von Willebrand factor. British Journal of Haematology. 2023;201(3):552–63.

128.	Choi SJ, Dwyer CN, Rapkin L, Cormier M, Hindmarch CCT, Nesbitt K, et al. The mechanistic and structural role of von Willebrand factor in endotoxemia-enhanced deep vein thrombosis in mice. Journal of Thrombosis and Haemostasis. 2023;21(3):586–98.

129.	De Wilde M, Desender L, Tersteeg C, Vanhoorelbeke K, De Meyer SF. Spatiotemporal profile of neutrophil extracellular trap formation in a mouse model of ischemic stroke. Research and Practice in Thrombosis and Haemostasis. 2023;7(1):100028.

130.	Shi H, Shao B, Gao L, Venkatesan T, McDaniel JM, Zhou M, et al. Endothelial VWF is critical for the pathogenesis of vaso-occlusive episode in a mouse model of sickle cell disease. Proceedings of the National Academy of Sciences of the United States of America. 2022;119(34):e2207592119.

131.	van Moorsel MVA, de Maat S, Vercruysse K, van Leeuwen EM, Jacqmarcq C, Bonnard T, et al. VWF-targeted thrombolysis to overcome rh-tPA resistance in experimental murine ischemic stroke models. Blood. 2022;140(26):2844–8.

132.	Globisch MA, Onyeogaziri FC, Jauhiainen S, Yau ACY, Orsenigo F, Conze LL, et al. Immunothrombosis and vascular heterogeneity in cerebral cavernous malformation. Blood. 2022;140(20):2154–69.

133.	Courson JA, Lam FW, Langlois KW, Rumbaut RE. Histone-stimulated platelet adhesion to mouse cremaster venules in vivo is dependent on von Willebrand factor. Microcirculation. 2022;29(8):e12782.

134.	Stivala S, Gobbato S, Bonetti N, Camici GG, Luscher TF, Beer JH. Dietary alpha-linolenic acid reduces platelet activation and collagen-mediated cell adhesion in sickle cell disease mice. Journal of Thrombosis and Haemostasis. 2022;20(2):375–86.

135.	Xu L, Qiu Y, Li Y, Wei Y, Wan Y, Deng W. Tissue dynamics of von Willebrand factor characterized by a novel fluorescent protein-von Willebrand factor chimera. Journal of Thrombosis and Haemostasis. 2022;20(1):208–21.

136.	Shao B, Hoover C, Shi H, Kondo Y, Lee RH, Chen J, et al. Deletion of platelet CLEC-2 decreases GPIbalpha-mediated integrin alphaIIbbeta3 activation and decreases thrombosis in TTP. Blood. 2022;139(16):2523–33.

137.	South K, Saleh O, Lemarchand E, Coutts G, Smith CJ, Schiessl I, et al. Robust thrombolytic and anti-inflammatory action of a constitutively active ADAMTS13 variant in murine stroke models. Blood. 2022;139(10):1575–87.

138.	Heger LA, Schommer N, Van Bruggen S, Sheehy CE, Chan W, Wagner DD. Neutrophil NLRP3 promotes cardiac injury following acute myocardial infarction through IL-1β production, VWF release and NET deposition in the myocardium. SCIENTIFIC REPORTS. 2024;14(1).

139.	Sun TH, Liu J, Yan TS, Chen AJ, Zhang F. Kelp Fucoidans Facilitate Vascular Recanalization <i>via</i> Inhibiting Excessive Activation of Platelet in Deep Venous Thrombosis Model of Mouse. JOURNAL OF OCEAN UNIVERSITY OF CHINA. 2024;23(2):550–6.

140.	Schoner A, Tyrrell C, Wu M, Gelow JM, Hayes AA, Lindner JR, et al. Endocardial Endothelial Dysfunction Progressively Disrupts Initially Anti then Pro-Thrombotic Pathways in Heart Failure Mice. PloS one. 2015;10(11):e0142940.

141.	Dmitrieva NI, Burg MB. Secretion of von Willebrand factor by endothelial cells links sodium to hypercoagulability and thrombosis. Proceedings of the National Academy of Sciences of the United States of America. 2014;111(17):6485–90.

142.	Jongejan YK, Linthorst NA, Schrader Echeverri E, Laan SNJ, Dirven RJ, Dahlman JE, et al. Impact of allele-selective silencing of von Willebrand factor in mice based on a single nucleotide allelic difference in von Willebrand factor. Thrombosis Research. 2024;236((Jongejan, Linthorst, Laan, Dirven, van Vlijmen, Eikenboom) Department of Internal Medicine, Division of Thrombosis and Hemostasis, Einthoven Laboratory for Vascular and Regenerative Medicine, Leiden University Medical Center, Leiden, Netherlands(Schrader):201–8.

143.	Peetermans M, Meyers S, Liesenborghs L, Vanhoorelbeke K, De Meyer SF, Vandenbriele C, et al. Von Willebrand factor and ADAMTS13 impact on the outcome of Staphylococcus aureus sepsis. J Thromb Haemost. 2020;18(3):722–31.
